# Cost and Affordability of Habitual and Recommended Diets in Welfare-Dependent Households in Australia

**DOI:** 10.3390/nu16050659

**Published:** 2024-02-26

**Authors:** Meron Lewis, Stephen Nash, Amanda J. Lee

**Affiliations:** School of Public Health, Faculty of Medicine, The University of Queensland, Herston, QLD 4006, Australia; stephen.nash@uq.net.au (S.N.); amanda.lee@uq.edu.au (A.J.L.)

**Keywords:** diet cost, diet affordability, food insecurity, food price, low socioeconomic, low income, Australia

## Abstract

It is crucial to ensure healthy diets are affordable in low socioeconomic groups, such as welfare-dependent households, who experience higher rates of diet-related disease than others. This study assessed the cost of habitual (unhealthy) and recommended (healthy) diets in six welfare-dependent and six other, comparable Australian households, using either popular branded products or the cheapest available alternatives. It also assessed diet affordability in welfare-dependent households, before and after modest increases in government welfare payments introduced in early September 2023. Results confirmed that recommended diets were less expensive than habitual diets in all households unless the cheapest available products were included. This strategy reduced habitual diet costs by 35–37% and recommended diet costs by 30–32%. The lower cost differential could aid perceptions that healthy foods are more expensive than unhealthy foods. In April 2023, 23–37% of the income of welfare-dependent households with children was required to purchase recommended diets; this reduced only to 20–35% in September 2023. Hence, the increases in welfare payments were insufficient to meaningfully improve the affordability of healthy diets in the most vulnerable Australians. In the current cost-of-living crisis, there is an urgent need for more welfare support to help purchase healthy diets. Monitoring of diet cost and affordability is also required.

## 1. Introduction

Poor diet is a key contributor to the burden of disease globally and in Australia, being a major risk factor for many non-communicable diseases (NCDs) such as cardiovascular disease, type 2 diabetes, and some cancers [1,2]. The Australian Dietary Guidelines (ADGs) [3,4] provide dietary recommendations to reduce the risk of NCDs, yet less than 4% of Australians consume a diet in line with these [5]. A key recommendation of the ADGs is to minimise the consumption of “discretionary foods”, that is, food and drinks which are not required for health and are high in saturated fat, added sugar, salt and/or alcohol [3]. However, more than a third of adults’, and more than 40% of children’s, energy intakes are derived from discretionary food and drinks [5].

As in many high-income countries, rates of diet-related disease in low socioeconomic groups (SEGs) are higher than in the broader Australian population [6,7,8]. Reported dietary intakes of low SEGs score lower in nutritional quality compared to those of higher SEGs [9] as they contain less healthy foods but similar intakes of discretionary foods [10]. Price is a commonly cited determinant of food choice, and the affordability of healthy food is a key factor in the inequities of healthy eating [11,12,13]. The relative cost of healthy and unhealthy food and drinks within the context of dietary patterns, is an important leverage point to target to encourage the consumption of healthy diets [11].

Consistent with the optimal approach developed by the International Network for Food and Obesity/Non-Communicable Diseases Research Monitoring and Action Support (INFORMAS) group [14], the Healthy Diets Australian Standardised Affordability and Pricing (HD ASAP) protocol was developed to assess, monitor and compare the cost, cost differential and affordability of habitual and recommended diets for the mean population in Australia [15]. The successful application of the HD ASAP protocol in various locations across Australia has challenged the common perception that healthy diets are more expensive than habitually consumed diets [15,16,17]. Results were found and similar conclusions drawn in Mexico [18] and New Zealand [19]. The HD ASAP studies relate to the mean reported dietary intakes of the whole population and include popular, commonly available major national/international brands (“popular brands”) of foods and drinks [15,16,17]. However, low-income households frequently stretch their food budget by implementing strategies such as purchasing generic brands (also known as “own brands”, “private labels” or “home brands”) and using discount supermarkets [20,21,22]. To accommodate these strategies, a modified version of the HD ASAP protocol was developed to reflect the needs and lived experience of low SEGs [23]. The classification of SEG can be based on several metrics, such as income, education, area of residence, and occupation. As it reflects the household resources to purchase food, the metric of household income in the lowest quintile for Australia was used to classify the lowest SEG in the modified HD ASAP protocol [23]. The development and testing of the modified low SEG HD ASAP protocol has been published previously [23]. The current cost-of-living crisis [24] has led to rapidly rising food prices. The posting of large profits by the major supermarket chains over the same timeframe has led to current national [25] and state [26] inquiries into supermarket price-setting practices, market power, and supplier dealings by the supermarkets in Australia. In the light of these issues, a broader application of the modified low SEG HD ASAP protocol was warranted to better assess the cost and affordability of healthy diets in low SEGs.

Two thirds of the household income in the lowest income quintile in Australia is provided by government pensions and allowances [27]. The rate of unemployment benefits in Australia is one of the lowest in the Organisation for Economic Co-operation and Development (OECD) [28] and many calls have been made for its increase, particularly in recent years [28,29,30,31]. On 20 September, 2023, the Australian Government increased various welfare payments, including the unemployment benefit and rental assistance payments, by a modest amount and altered some eligibility requirements [32].

Doubling of the unemployment benefit payment during the early days of the COVID-19 pandemic in 2020 in Australia rendered healthy diets affordable in many welfare-dependent households for the first time [33]. However, the additional payments were soon reduced, and then removed completely in March 2021 [33]. Continued investigations of the impact of welfare policies on food affordability are critical for exploring the inequities faced by those reliant on such benefits.

The aims of this study were to assess the cost of habitual and recommended diets in welfare-dependent and other Australian households, using either popular branded products or the cheapest available alternatives. It also aimed to assess diet affordability in welfare-dependent households before and after the modest increases in government welfare payments introduced in early September 2023.

## 2. Materials and Methods

### 2.1. Household Composition

The whole of population and modified low SEG HD ASAP methods [15,23] provide the details and rationale for the choice of the six common household compositions studied (Table 1). These are coded Household A to Household F in Table 1.

### 2.2. Randomisation of Locations and Stores

The same locations were included as in previous surveys in Brisbane in order to support comparison of costs over time [31]. Statistical Area 2 (SA2) locations across Greater Brisbane had been stratified according to the Socioeconomic Indexes for Areas (SEIFA) Index of Relative Socioeconomic Disadvantage (IRSD) and nine were randomly sampled. The sample included three locations each from quintile 1 (most disadvantaged), quintile 3 (median disadvantaged), and quintile 5 (least disadvantaged) areas.

In each location, for the median households, food prices were collected from two large supermarkets (one of each major Australian supermarket chain), an independent grocery store, relevant take-away stores, and a liquor store. The same stores were usually surveyed as for the previous survey in 2021 [17] unless a store had closed, when a similar store in the same location area was included. For the low SEG households, food prices were collected from the same two large supermarkets, burger chain restaurant, and liquor store, but a discount supermarket was included in place of the independent grocery store. To reflect the reported lower spending on food from restaurants and takeaways by low SEGs in surveys of household expenditure [32], the prices of three frozen food items (a plain beef pie, hot chips, and a “supreme” pizza), were collected from relevant supermarkets instead of those of hot items from takeaway stores. In total, 81 stores across the nine locations were surveyed for food and drink prices.

### 2.3. Calculation of Household Income

Household incomes were calculated for each welfare-dependent household, using the payment rules and data from Services Australia in April and September 2023 [34] informed by a set of assumptions of the household’s situation (Table 1).

In April 2023, welfare payments included in household income were unemployment benefit for the adults looking for work in Households A, B, C, and E; parenting payment for the not-working adult in Household A; and age pension for the older adults in Households A, D, and F. In September 2023, the same welfare payments as April 2023 were included for Households A, B, and D–F. For Household C, the higher “single parent payment” was included rather than the unemployment benefit, due to a change in eligibility [32]. At both timepoints, rental assistance for households paying private rent, and family benefits for households with children were included.

### 2.4. Assessment of Diet Cost and Affordability

In April 2023, the cost and cost differential of habitual and recommended diets were assessed in the Greater Brisbane region of Queensland, Australia, in median households, by application of the original HD ASAP protocol [15], and in welfare-dependent households by application of the modified low SEG HD ASAP protocol [23]. All costs were reported in Australian dollars (AUD). Affordability of recommended diets were assessed in welfare-dependent households using the low SEG HD ASAP methods [23].

#### 2.4.1. Diet Pricing Tools

The diet-pricing tools specify the types and quantities of foods and drinks to include. The habitual diet-pricing tool of the whole of population HD ASAP protocol [15] is based on mean reported dietary intakes of relevant respondents in the National Health Survey National Nutrition and Physical Activity Survey (AHS NNPAS) of 2011–2013 [35]. The habitual diet-pricing tool of the low SEG HD ASAP protocol includes the same types of foods and drinks (Table 2); however, quantities reflect the mean reported intakes of AHS NNPAS respondents whose household income was in the lowest quintile [23]. Both habitual diets include some healthy food and drinks, in amounts lower than the recommendations of the ADGs, and a large quantity of discretionary food and drinks.

The recommended diet-pricing tool is based upon healthy food and drinks in quantities corresponding to the recommendations of the ADGs [3]. As the dietary guidelines are the same for all socioeconomic groups, the recommended diet-pricing tool which is informed by the minimum change from habitual diet to meet the guidelines is the same for both the median and welfare-dependent households. Detailed lists of the type and quantity of food and drink items included in the habitual and recommended diet-pricing tools for each household in this study are included as Supplementary Tables S1–S6 and the development of the diets was previously described in the HD ASAP protocol papers [15,23].

#### 2.4.2. Collection of Food and Drink Prices

Prices were collected by a trained research assistant (S.N.), in April 2023, following the price collection protocols of the HD ASAP methods [15,23], for example, not including collection of “specials” or price promotions. Permission to collect data was obtained from national head offices of the large supermarkets and the discount supermarket chain, and from store managers of each outlet visited instore. For the large supermarkets and alcohol outlets (27 stores), prices were collected from the stores’ online websites, where the shopping or “pickup” location was selected to match each survey location, as online prices and in-store prices have previously been shown to be comparable [22]. Prices were collected instore for the other 54 stores. Price data were entered into the HD ASAP web portal [36] by S.N. and checked by M.L.

Of the 76 food and drink items surveyed, 59 were packaged items where popular brands were specified in the price collection tool. To assess diet costs in welfare-dependent households, the prices of the cheapest available packaged food and drinks similar in nature to the specified products were also collected. The cheapest product was determined as that with the lowest unit price (i.e., AUD/g) of all similar products. If a similar sized product with a lower unit price was not available, products were selected that were a larger size than the specified product but had a lower unit price and a total price that was not more than AUD 1 more than the specified product.

For estimation of diet cost in September 2023, an adjustment factor consistent with the change in Consumer Price Index (food) (CPI (food)) in Brisbane between April and September 2023 for each food group was applied to each item price [37].

### 2.5. Data Entry, Analysis and Reporting

Price data were entered in the HD ASAP web portal by S.N. [36]. Data were cleaned and checked by M.L. If a value was missing, the mean price of the item in other stores in the same location was substituted. Spreadsheet algorithms generated results for each location in Microsoft Office Excel files, which were cross-checked by M.L. and S.N.

Habitual and recommended diet costs were calculated for each household type in each location using the prices of popular branded products for both the median and welfare-dependent households and also using the prices of the cheapest option products for welfare-dependent households. The mean costs of the habitual and recommended diets, and the cost and proportion of the total spent on different ADG food groups and components, were calculated for each household per fortnight. Results were reported for Greater Brisbane as a whole, similar to relevant previous studies [17].

Affordability of recommended diets was calculated for each welfare-dependent household as the proportion of household income required to purchase the diet. This was calculated for diet costs using either the prices of the popular branded or cheapest available products. Diets were considered unaffordable if the diet cost was 30% or more of household income [38], and as causing “food stress” if the diet cost required 25–30% of household income [39].

## 3. Results

### 3.1. Cost of Habitual and Recommended Diets

Food and drink prices were collected from nine locations, including both popular branded products and the cheapest option equivalents. For most packaged items (*n* = 52/59, 88%), a clearly equivalent item from a generic brand was the cheapest.

**Figure 1:**
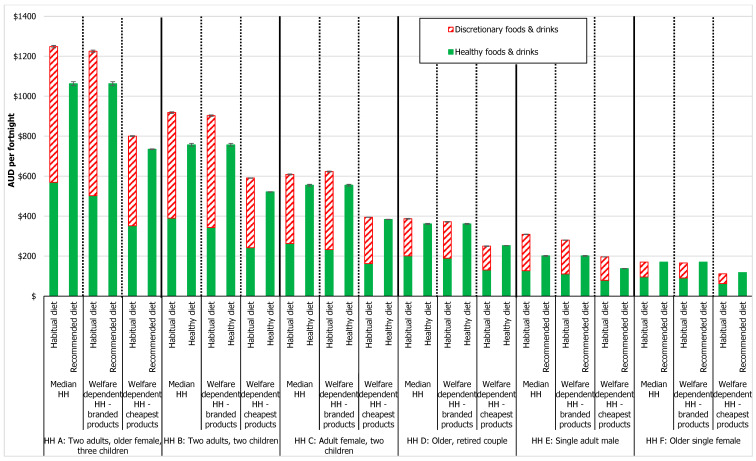
Cost of habitual and recommended diets in six household compositions (HH = household).

The costs of the habitual and recommended diets in the median and welfare-dependent households in April 2023 are shown in Figure 1. Detailed costs by food group are provided in Supplementary Tables S7–S12. Total diet and food group costs calculated in September 2023 are provided in Supplementary Tables S13–S19. Total diet costs increased by around 1.27% from April to September 2023 [37].

#### 3.1.1. Diet Cost in Median Households

In most median households using the popular brands, the recommended diet was 7–53% less expensive than the habitual diet (Figure 1), except in Household F where the diets costs were equivalent. The largest differential between habitual and recommended diet costs was in Household E, for whom the recommended diet would cost 53%, or AUD 108/fortnight, less than the habitual diet. The recommended diet was less expensive than the habitual diet in Household A (AUD 186/fortnight, 17% less), Household B (AUD 161/fortnight, 21% less), Household C (AUD 54/fortnight, 10% less), and Household D (AUD 26/fortnight, 7% less).

As expected, household diet costs increased with increasing household size. The larger difference between the recommended and habitual diet cost in Household E (single adult male) compared to the other household compositions was due to the higher reported intake of discretionary products, especially alcohol, in adult males aged 31–50 y compared to other age and gender groups. The higher discretionary product intake of adult males also led to the larger differential between habitual and recommended diet costs in the two parent (adult male and adult female) Household B compared to the single parent (adult female) Household C.

#### 3.1.2. Diet Cost in Welfare-Dependent Households Using Popular Brands

In most welfare-dependent households using the popular brands, the recommended diet was 3–39% less expensive than the habitual diet, except in Household F where the recommended diet was more expensive (AUD 7/fortnight, 6% more).

##### Comparison of Diet Cost in Median and Welfare-Dependent Households

Compared to median households, the cost of the habitual diet in welfare-dependent households using popular branded products, was 3% less (9% less to 2% more). The recommended diet-pricing tool was the same in both median and welfare-dependent households, and thus recommended diets were of equivalent cost.

In welfare-dependent households overall, healthy items within the habitual diet cost 10% less (6–13% less) than in median households. Discretionary items cost 3% more (1% less to 13% more) in welfare-dependent households than in median households. More specifically, in welfare-dependent households with children (Households A, B, and C) the cost of healthy items in the habitual diet was 12% less (same in all household compositions) and the cost of discretionary items was 8% (6–13%) more than in median Households A, B and C.

#### 3.1.3. Diet Cost in Welfare-Dependent Households Using Cheapest Available Products

In most welfare-dependent households using the cheapest available products, the recommended diet was 9–42% less expensive than the habitual diet. Exceptions where the recommended diet was slightly more expensive than the habitual diet were in Household D (AUD 3/fortnight, 1% more) and Household F (AUD 7/fortnight, 6% more).

The largest differential between habitual and recommended diet costs was in Household E, for whom the recommended diet would cost 42%, or AUD 59/fortnight, less than the habitual diet. The recommended diet was less expensive than the habitual diet in Household A (AUD 65/fortnight, 9% less), Household B (AUD 69/fortnight, 13% less), and Household C (AUD 11/fortnight, 3% less).

##### Comparison of Diet Cost Using Popular Brands and Cheapest Available Products

In welfare-dependent households, the habitual and recommended diet costs were 34% (31–37%) and 31% (30–35%) lower, respectively, using the cheapest available products compared to using the popular brands. Thus, the cost differential between the habitual and recommended diet cost was smaller when using the cheapest options compared to popular brands (Figure 1).

#### 3.1.4. Proportion of Habitual Diet Cost Spent on Discretionary Foods and Drinks

Across all household compositions, the cost of discretionary foods and drinks made up a large proportion, 43–63%, of the total habitual diet costs.

Discretionary foods and drinks made up the majority, 54–63%, of total habitual diet costs in the households with children (Households A, B, C), and the single adult male household (Household E). The proportion was slightly less in the older person households, Household D (48–49%) and Household F (43–45%), reflecting a lower consumption of these foods in older age groups, particularly older women [10].

### 3.2. Diet Affordability in Welfare-Dependent Households

#### 3.2.1. Household Income

Table 3 presents the incomes of the six reference welfare-dependent household compositions per fortnight in April 2023 and after welfare changes in September 2023. Detailed calculations of the welfare-dependent household incomes are provided in Supplementary Table S19.

Increases in household income in September 2023 were minimal in the older person households, Household D (AUD 75/fortnight (4.3%)) and Household F (AUD 61/fortnight (4.9%). The income of Households A, B, and E increased by AUD 207/fortnight (6.5%), AUD 185/fortnight (9.1%), and AUD 89 (11.4%), respectively. Household C experienced an income increase of AUD 310/fortnight (19.5%).

#### 3.2.2. Affordability of the Recommended Diet in Welfare-Dependent Households

**Figure 2:**
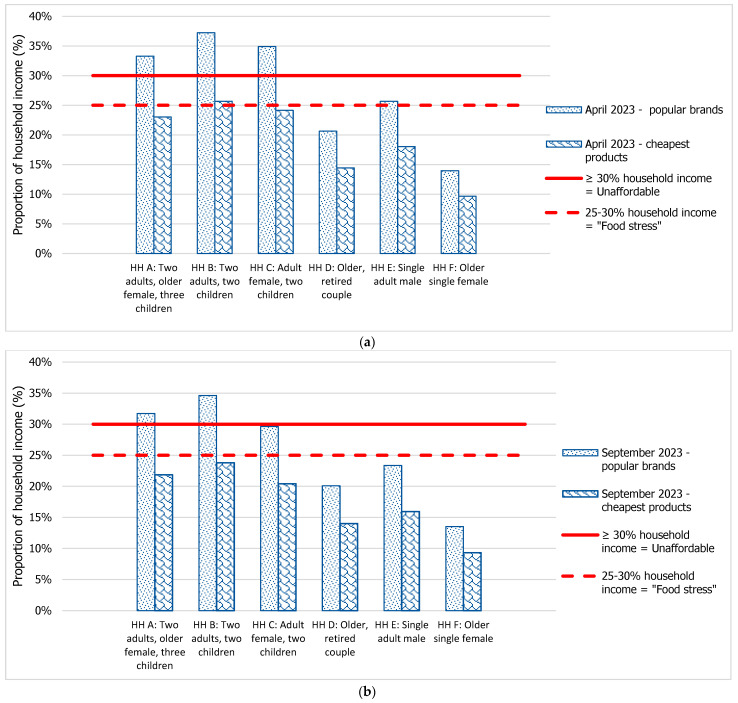
(**a**) Affordability of the recommended diet using popular branded products or cheapest products (% household income) in six welfare-dependent household compositions, in April 2023 (HH = household). (**b**) Affordability of the recommended diet using popular branded products or the cheapest products (% household income) in six welfare-dependent household compositions, in September 2023 (HH = household).

The affordability of the recommended diet in welfare-dependent households, costed using popular branded products or the cheapest options, is shown in Figure 2a (April 2023) and Figure 2b (September 2023).

In April 2023, the recommended diet was unaffordable when purchasing the popular branded products in Household A (33.3% of household income), Household B (37.2% of household income), and Household C (34.9% of household income) (Figure 2a). The recommended diet was stressful to afford in Household E (25.7% of household income) but was affordable in Household D (20.6% of household income), and Household F (14.0% of household income).

In April 2023, when the cheapest options were purchased, the recommended diet was still stressful to afford in Household B (25.6% of household income), and close to stressful to afford in Household A (23.0% of household income), and Household B (24.1% of household income) (Figure 2a). The recommended diet was affordable in Households D, E and F (14.4%, 18.0%, and 9.7% of household income, respectively).

When calculated in September 2023, minimal improvements in the affordability of a recommended diet were observed in welfare-dependent households (Figure 2b). The affordability of recommended diets when pricing popular brands slightly improved by 0.6% in Household D, 0.4% in Household F, and by 1.6%, 2.3% and 2.6% in Households A, E and B, respectively. Household C experienced a greater improvement in recommended diet affordability of 5.3%.

Similarly, the affordability of recommended diets in September 2023 when pricing the cheapest options improved by 0.4% in Household D, 0.3% in Household F, and by 1.2%, 2.1% and 1.9% in Households A, E and B, respectively. Household C experienced an improvement in recommended diet affordability of 3.7% (Figure 2b).

The increased income in September 2023 was sufficient to move Household C just out of “unaffordability” then requiring 29.6% of household income to purchase a recommended diet using popular brands, but the household remained at risk of food stress unless the cheapest options were always selected (20.4% of household income). Similarly, recommended diets with popular brands remained unaffordable in Household A (31.7% of household income) and Household B (34.6% of household income) unless the cheapest options were always selected (21.9% and 23.8% of household income, respectively).

## 4. Discussion

### 4.1. Cost of Habitual and Recommended Diets

This study assessed the cost of habitual and recommended diets in welfare-dependent and median Australian households, using either popular branded products or the cheapest available alternatives. We found that recommended diets were less expensive than habitual diets for all households and pricing options, apart from some older person households. This pattern is consistent with previous studies using the HD ASAP and the low SEG HD ASAP approach [16,17,23].

The exemption of basic, healthy foods from the 10% Good and Services Tax (GST) in Australia contributes to the relatively lower cost of the recommended diet in most households. Efforts to maintain the GST exemption and promote the consumption of recommended diets can aid households to save money and improve health. Additionally, recommended diets are more sustainable, requiring less water, protecting biodiversity, and generating 25% lower greenhouse gas emissions in their production compared to habitual diets [40].

Habitual diets in this study include 43–63% of the cost attributable to discretionary foods and drinks, which include alcohol and takeaway foods. The higher cost of habitual diets compared to recommended diets further supports the evidence that while price may be an important determinant of food choice, other influences are also strong. These include determinants such as taste, time and convenience (shopping and preparation), nutrition/healthiness, societal influence, accessibility, and the packaging, advertising, marketing and promotion of unhealthy foods and drinks [16].

Total habitual diet costs were similar in median and welfare-dependent households. However, in welfare-dependent households, the proportion of the habitual diet cost spent on healthy food and drinks was lower, and was higher for discretionary food and drinks. These results show the same pattern as the findings of a pilot study [23], and reflect the differing reported dietary intakes between the population groups. The most recent Household Expenditure Survey (2015–2016) reported expenditure on all food and drinks (including alcohol) was AUD 312/fortnight for the lowest income quintile, AUD 794/fortnight for the highest income quintile, with a mean of AUD 538/fortnight for all households [41]. However, these data provide mean expenditure for all Australian households. Differences in spending may be due to differences in household size, food choice, brand choice and dietary intake.

### 4.2. Effect of Purchasing the Cheapest Available Option

As expected, when welfare-dependent households chose the cheapest options available at major and discount supermarkets, meaningful cost savings ensued. The savings were greater for the habitual diet (34%) than for the recommended diet (31%). These differences arose due to more packaged products being included in the former than the latter. This resulted in the cost of recommended diets becoming relatively more expensive compared to habitual diets when cheapest options were chosen.

The reduced cost differential between habitual and recommended diets may help explain the common perception that recommended diets are too expensive. However, the increased availability of low-priced generic versions of packaged, usually ultraprocessed, foods may encourage their purchase.

While low SEGs are more likely to purchase generic brands [20] than popular brands, some low SEGs may experience social stigma if purchasing only generic brands [21]. Qualitative studies of the shopping habits of low-income households have also revealed that some people are loyal to the purchase of popular brands [42]. The reasons given include that popular brands are considered to be trustworthy, and their purchase reduces the risk of the product being wasted if not suitable for the household’s taste and requirements [42]. Additionally, in more regional and remote locations, discount supermarkets are not available and/or stores are smaller independent stores which may have reduced access to generic brands. Therefore, it could be more realistic for advocacy purposes to include a range or mid-point between diet costs generated using popular brands and those using cheapest options [43].

### 4.3. Diet Affordability before and after Changes in Welfare

The results for the six welfare-dependent households show that the increased welfare payments in September 2023 resulted in only small improvements in recommended diet affordability (0.4–5.3%) compared to April 2023. Recommended diets remained unaffordable for most household structures. Although the largest improvement (5.3%) was shown in Household C (adult female, two children), recommended diets were still stressful to afford for this household in September 2023 unless most items purchased were the cheapest available option. In addition, this improvement needs to be considered in the context of continued increases in housing, fuel, utilities and other costs of living, as evidenced by a 13% increase in CPI since the last welfare payment increase in March 2021 [37]. These increases likely mean that the proportion of the household budget available for purchasing food has reduced in recent times, such that any improvement in food affordability has been utilised for other household costs. The definition of diet affordability as 30% of household income is in common usage in the literature, but is an arbitrary number based on the concept of one third of household income being assigned to housing, one third to food, and one third for remaining expenses [38]. Media reports in 2023 suggested that low-income households were spending 50% of their income on housing [44], leaving such households with a limited budget to spend on healthy food.

Calls to increase welfare payments have been made for many years in Australia, particularly focused on unemployment benefits, to help ensure healthy food and other essential goods and services, including housing, are affordable [45]. The increase in welfare payments in September 2023 were described as “cost-of-living relief measures” and acknowledged to be a “modest increase” [46], exemplified by the increase in income of 9.1% in the two adult, two child welfare-dependent household. In comparison, the provision of additional payments through the welfare system during the early months of the COVID-19 pandemic increased the income of a two adult, two child welfare-dependent household by 77% [17]. Welfare-dependent households were then able to afford a healthy diet and a survey at that time found many families were purchasing an increased amount of healthy food as a result [33].

The determination of diet cost and affordability for welfare-dependent households, using the methods of this study, has been included in advocacy reports assessing the cost of living that show low-income households struggle to meet basic standards of living and are at increased risk of food insecurity [43]. Continued monitoring of data is critical to maintain pressure on governments to ensure that welfare support is adequate to allow households to afford a healthy diet. The tools and protocols used in this study are available for use in Australia, upon application to the authors.

### 4.4. Differences in Diet Cost and Affordability in Households of Different Compositions

Older adults report dietary intakes with lower amounts of discretionary food and drinks than other groups [10]. This may reflect shopping and cooking habits from earlier in their life when convenience and takeaway foods were less available [47]. This resulted in the lower cost differential between the habitual and recommended diets in the older, retired couple and older, single female household compositions than in the other households.

The affordability of the recommended diet in welfare-dependent older person households was more attainable than in the other households studied. One reason for this is the higher income per person in households receiving the age pension compared to households receiving unemployment benefits. In Australia, the age pension is indexed to average wages, unlike unemployment benefits which are indexed to inflation. This has contributed to higher increases for the age pension compared to unemployment benefits over recent decades.

Unemployment benefits did not increase in real terms between 2009 and 2020 [30]. This study has identified that the affordability of recommended diets was worst in households with children reliant on these payments. The welfare changes in September 2023 did little to improve affordability of recommended diets for most households. One exception was those households who benefited from changes in eligibility to the single parent pension, resulting in a higher payment rate. Support for families to purchase and consume healthy diets is particularly important for the future health and wellbeing of children [3].

### 4.5. Limitations

The limitations of the whole of population and low SEG HD ASAP methods and protocols have been discussed elsewhere [15,23]. This study focused on Greater Brisbane and may not be generalizable particularly in rural and remote areas which have much higher food prices [16,48]. Healthy diets are less affordable in these areas [16].

## 5. Conclusions

It is critical that the GST exemption on basic healthy foods in Australia is maintained to keep recommended diets as affordable as possible for welfare-dependent households. It is also important that the common perception that healthy food is more expensive than unhealthy foods is countered, to encourage healthier diets and reduce the risk of diet-related disease.

The current cost-of-living crisis has prompted national [25] and state [26] inquiries into supermarket prices in Australia. It is essential that these inquiries consider the impact of the cost of healthy foods and drinks for welfare-dependent households.

In the context of rapidly increasing household expenses since 2021, the risk of food insecurity in many low-income households remains. Further increases in welfare payments are required to ameliorate this risk.

If recommended diets were affordable for all, this would lead to improved workforce and social participation, improved education outcomes for children, reduced future health costs and reduced social inequality. The recently released National Preventive Health Strategy includes the goal, by 2030, of ensuring “ongoing access to adequate and affordable healthy food options [for] all Australians” [49]. To achieve this goal, continued monitoring and surveillance of healthy diet costs are required to produce real-world data for use in advocacy efforts to continue pressuring governments to provide sufficient welfare support to promote the purchase of a healthy diet.

## Figures and Tables

**Table 1:** Composition of households and assumptions used to calculate welfare-dependent household income.

Household Structure	Two Adults, Older Female, Three Children (Household A)	Adult Male and Female, Two Children (Household B)	Adult Female, Two Children (Household C)	Older, Retired Couple (Household D)	Single Adult Male (Household E)	Older Single Female (Household F)
Median households	Female (31–50 y) Male (31–50 y) Older female (70+ y) Boy (14 y) Girl (8 y) Boy (4 y)	Female (31–50 y) Male (31–50 y) Boy (14 y) Girl (8 y)	Female (31–50 y) Boy (14 y) Girl (8 y)	Older female (70+ y) Older male (70+ y)	Male (31–50 y)	Older female, (70+ y)
Welfare-dependent households	Female (31–50 y) Male (31–50 y) Older female (70+ y) Boy (14–18 y *) Child (4–8 y *) Child (4–8 y *)	Female (31–50 y) Male (31–50 y) Boy (14–18 y *) Child (4–8 y *)	Female (31–50 y) Boy (14–18 y *) Child (4–8 y *)	Older female (70+ y) Older male (70+ y)	Male (31–50 y)	Older female, (70+ y)
Assumptions used to calculate welfare-dependent income	• Adult male is unemployed and looking for work • Adult female is a stay-at-home parent • Older female receives full age pension (maximum rate) • The children attend school/kindy • None of the family are disabled • No savings or investments • The family is living in public housing	• Both adults are unemployed and looking for work • Both children attend school • None of the family are disabled • No savings or investments • Private rent at AUD 379/week	• Adult female is unemployed and looking for work • No child support received • Both children attend school • None of the family are disabled • No savings or investments • Private rent at AUD 379/week	• Neither are in paid employment • Both receive the full age pension (maximum rate) • Neither are disabled or frail-aged • The couple has no dependent children • Private rent at AUD 379/week	• Is unemployed and looking for work • Is not disabled • No dependent children • No savings or investments • Is renting a room in 3-bedroom house at AUD 125/week (AUD 376/3)	• Not in paid employment • Receives full age pension (maximum rate) • Not disabled or frail-aged • Has no dependent children • Private rent at AUD 379/week

* Adjusted for sample size in Australian Health Survey National Nutrition and Physical Activity Survey (AHS NNPAS) 2011-13 (29). y = years.

**Table 2:** Food and drinks included in the original (median population) and modified low SEG HD ASAP diet-pricing tools.

Habitual (Unhealthy) Diet	Recommended (Healthy) Diet
Healthy foods and drinks as per the seven food groups in the “Recommended diet” column; in reduced amounts reflecting reported intake. Artificially sweetened beverages. Discretionary (unhealthy) foods and drinks: Drinks: sugar sweetened beverages. Cereals, snacks, and desserts: muffin, sweet biscuits, savoury crackers, confectionery, chocolate, potato crisps, muesli bar, mixed nuts (salted), ice cream, fruit salad (canned in juice). Processed meats: beef sausages, ham. Spreads, sauces, condiments, and ingredients: butter, tomato sauce, salad dressing, white sugar. Convenience meals: frozen lasagne, chicken soup (canned), frozen fish fillet (crumbed), instant noodles, meat and vegetable stew (canned). Fast food: pizza, meat pie, hamburger, potato chips/fries. Alcohol: beer (full strength), white wine (sparkling), red wine, whisky.	Water (bottled). Fruit: apples, bananas, oranges. Vegetables: potatoes, broccoli, white cabbage, iceberg lettuce, onion, carrot, pumpkin, tomatoes, sweetcorn (canned), four bean mix (canned), diced tomatoes (canned), baked beans (canned), frozen mixed vegetables, frozen peas, salad vegetables in sandwiches. Grain (cereals): wholegrain cereal biscuits (Weet-bix™), rolled oats, cornflakes, wholemeal bread, white bread, white rice, white pasta, dry water crackers, bread in sandwiches. Lean meats and alternatives: beef mince and steak, lamb chops, cooked chicken, tuna (canned), eggs, peanuts (unsalted), meat in sandwiches. Milk, yoghurt, and cheese: cheddar cheese (full fat, reduced fat), milk (full fat, reduced fat), yoghurt (full fat plain, reduced fat flavoured). Unsaturated oils and spreads: olive oil, sunflower oil, canola (margarine).

**Table 3:** Fortnightly household income (AUD) in six welfare-dependent household compositions, in April 2023 and September 2023.

Household	A: Two Adults, Older Female, Three Children	B: Two Adults, Two Children	C: Adult Female, Two Children	D: Older, Retired Couple	E: Single Adult Male	F: Older Single Female
Fortnightly income—April 2023	AUD3192	AUD 2033	AUD 1591	AUD 1752	AUD 786	AUD 1221
Fortnightly income—September 2023	AUD 3400	AUD 2218	AUD 1901	AUD 1827	AUD 875	AUD 1282

## Data Availability

The data presented in this study are available in the text and Supplementary Material.

## Supplementary Materials

The following supporting information can be downloaded at: https://www.mdpi.com/article/10.3390/nu16050659/s1, Table S1: Composition of the original Healthy Diets ASAP habitual diet pricing tool for mean Australian population and the Low SEG Healthy Diets ASAP habitual diet pricing tool, and recommended diet pricing tool for a two adult, older female, three children household (Household A); Table S2: Composition of the original Healthy Diets ASAP habitual diet pricing tool for mean Australian population and the Low SEG Healthy Diets ASAP habitual diet pricing tool, and recommended diet pricing tool, for a two adults, two children household (Household B); Table S3: Composition of the original Healthy Diets ASAP habitual diet pricing tool for mean Australian population and the Low SEG Healthy Diets ASAP habitual diet pricing tool, and recommended diet pricing tool, for an adult female, two children household (Household C); Table S4: Composition of the original Healthy Diets ASAP habitual diet pricing tool for mean Australian population and the Low SEG Healthy Diets ASAP habitual diet pricing tool, and recommended diet pricing tool for an older retired couple (Household D); Table S5: Composition of the original Healthy Diets ASAP habitual diet pricing tool for mean Australian population and the Low SEG Healthy Diets ASAP habitual diet pricing tool, and recommended diet pricing tool for a single adult male (Household E); Table S6: Composition of the original Healthy Diets ASAP habitual diet pricing tool for mean Australian population and the Low SEG Healthy Diets ASAP habitual diet pricing tool, and recommended diet pricing tool for an older single female (Household F); Table S7: Calculations of welfare-dependent fortnightly incomes for the six welfare-dependent households in April 2023 and September 2023; Table S8: Diet cost and affordability by food group and food group components of habitual and recommended diets for a two adult, senior female, three children household (Household A) in Greater Brisbane, April 2023; Table S9: Diet cost and affordability by food group and food group components of habitual and recommended diets for a two adult, two children household (Household B) in Greater Brisbane, April 2023; Table S10: Diet cost and affordability by food group and food group components of habitual and recommended diets for an adult female, two children household (Household C) in Greater Brisbane, April 2023; Table S11: Diet cost and affordability by food group and food group components of habitual and recommended diets for an older retired couple household (Household D) in Greater Brisbane, April 2023; Table S12: Diet cost and affordability by food group and food group components of habitual and recommended diets for a single adult male household (Household E) in Greater Brisbane, April 2023; Table S13: Diet cost and affordability by food group and food group components of habitual and recommended diets for an older single female household (Household F) in Greater Brisbane, April 2023; Table S14: Diet cost and affordability by food group and food group components of habitual and recommended diets for a two adult, senior female, three children household (Household A) in Greater Brisbane, September 2023; Table S15: Diet cost and affordability by food group and food group components of habitual and recommended diets for a two adult, two children household (Household B) in Greater Brisbane, September 2023; Table S16: Diet cost and affordability by food group and food group components of habitual and recommended diets for an adult female, two children household (Household C) in Greater Brisbane, September 2023; Table S17: Diet cost and affordability by food group and food group components of habitual and recommended diets for an older retired couple household (Household D) in Greater Brisbane, September 2023; Table S18: Diet cost and affordability by food group and food group components of habitual and recommended diets for a single adult male household (Household E) in Greater Brisbane, September 2023; Table S19: Diet cost and affordability by food group and food group components of habitual and recommended diets for an older single female household (Household F) in Greater Brisbane, September 2023.
