# Supplementary material for: Cost and Affordability of Habitual and Recommended Diets in Welfare-Dependent Households in Australia
Source: Nutrients. 2024 Feb 26;16(5):659. doi: 10.3390/nu16050659 (PMC10935407; doi:10.3390/nu16050659)
Supplement: Supplementary file 1 [file nutrients-16-00659-s001.zip › Supplementary Tables S1 to S6 Diet pricing tools for HH A to F.pdf]

Table S1: Composition of the original Healthy Diets ASAP habitual diet pricing tool for mean Australian population and the Low SEG Healthy Diets ASAP habitual diet pricing tool, and recommended diet pricing tool for a two adult, older female, three children household (Household A)

Food item	Habitual Diet (g/fortnight)		Recommended Diet (g/fortnight) Original & Low SEG Healthy Diets ASAP
	Original Healthy Diets ASAP	Low SEG Healthy Diets ASAP	
**Energy (kJ/day)**	46943 kJ	45651 kJ	46404 kJ
**Water**			
Water, bottled (mL)	5948	3943 (34% < Original)	5948
**Fruit**			
Apples (g)	5072	5439	7910
Bananas (g)	1741	1501	7910
Oranges (g)	2496	1552	7910
Fruit salad, canned in juice (g)	3819	2899	0
Total Fruit (g)	17700	15593 (12% < Original)	23730
**Vegetables and Legumes**			
Potato, loose (g)	2181	2690	2970
Broccoli, loose (g)	620	594	2170
White cabbage, loose (g)	331	276	2170
Iceberg lettuce, whole (g)	1071	950	2170
Carrot, loose (g)	1130	1019	3255
Pumpkin (g)	407	532	3255
Onion, loose (g)	124	155	2373
Tomatoes, loose (g)	712	943	2373
Sweetcorn, canned (g)	427	390	1485
Four bean mix, canned (g)	111	73	1380
Diced tomatoes, canned (g)	314	272	2373
Baked Beans, canned (g)	525	352	1380
Frozen mixed vegetables (g)	1744	1125	2373
Frozen peas (g)	419	511	2373
Total Vegetables and Legumes (g)	11143	10622 (5% < Original)	32366
**Grain (Cereal) Foods – Wholegrain and Refined**			
Wholemeal bread, pre-packaged (g)	1680	1620	5472
White bread, pre-packaged (g)	4215	4322	1133
Rolled oats (g)	1629	1384	8688
Breakfast cereal, corn flakes (g)	1019	775	850
Breakfast cereal, wheat biscuits (g)	569	338	2896

Food item	Habitual Diet (g/fortnight)		Recommended Diet (g/fortnight) Original & Low SEG Healthy Diets ASAP
	Original Healthy Diets ASAP	Low SEG Healthy Diets ASAP	
White pasta (g)	1683	1460	2571
White rice (g)	2207	2368	2571
Dry wheat crackers, water crackers (g)	369	165	991
Total Grain (Cereal) Foods (g)	13528	12634 (7% < Original)	25438
**Lean Meats and Poultry, Fish, Eggs, Nuts and Seeds**			
Tuna, canned in oil (g)	1480	1119	2675
Beef mince, lean (g)	395	256	1514
Lamb loin chops (g)	454	543	1516
Beef rump steak (g)	1336	1315	1519
Eggs (g)	1306	1201	3208
Chicken, cooked whole (g)	2332	1703	2137
Peanuts, roasted, unsalted (g)	0	0	960
Total Lean Meats and Poultry, Fish, Eggs, Nuts and Seeds (g)	8330	6876 (17% < Original)	13795
**Milk, Yoghurt, Cheese and Alternatives**			
Cheddar cheese, full fat (g)	976	954	1104
Cheddar cheese, reduced fat (g)	71	72	814
Milk, full fat (mL)	8967	10373	10157
Milk, reduced fat (mL)	4348	2915	18543
Flavoured milk (mL)	3169	2744	0
Yoghurt, full fat, plain (g)	315	168	4064
Yoghurt, flavoured reduced fat (g)	1568	1197	8050
Total Milk, Yoghurt, Cheese and Alternatives (g)	19414	18422 (5% < Original)	42732
**Unsaturated Oils and Spreads (or foods from which these are derived)**			
Sunflower oil (mL)	9	22	380
Olive oil (mL)	9	22	380
Canola margarine (g)	266	314	538
Total Unsaturated Oils and Spreads (g)	284	358 (26% > Original)	1298
**Discretionary Choices – other**			
Chicken soup, canned (g)	2368	3386	0
Muffin, commercial (g)	2125	1463	0

Food item	Habitual Diet (g/fortnight)		Recommended Diet (g/fortnight) Original & Low SEG Healthy Diets ASAP
	Original Healthy Diets ASAP	Low SEG Healthy Diets ASAP	
Instant noodles, wheat based (g)	621	378	0
White sugar (g)	935	1126	0
Cream-filled sweet biscuit, pre-packaged (g)	807	992	0
Muesli bar, pre-packaged (g)	495	505	0
Savoury flavoured crackers (g)	262	278	0
Nuts, mixed, salted (g)	336	325	0
Confectionary (g)	625	605	0
Chocolate (g)	528	496	0
Potato crisps, pre-packaged (g)	809	865	0
Salad dressing (g)	497	319	0
Tomato sauce (g)	731	685	0
Beef sausages (g)	1459	1505	0
Butter (g)	455	266	0
Ham (g)	282	223	0
Frozen lasagne, pre-packaged (g)	5369	4579	0
Fish fillet crumbed, pre-packaged (g)	548	650	0
Ice cream (g)	2666	2066	0
Total Discretionary – other (g)	26489	24912 (6% < Original)	0
**Alcoholic Drinks**			
Beer, full strength (mL)	4905	5469	0
White wine, sparkling (mL)	1377	970	0
Whisky (mL)	356	572	0
Red wine (mL)	1385	788	0
Total Alcoholic Drinks (mL)	8023	7799 (3% < Original)	0
**Takeaway foods**			
Pizza, commercial (g)	1448	2242	0
Meat pie, commercial (g)	1982	1952	0
Hamburger, commercial (g)	2793	3272	0
Potato chips, commercial (g)	807	1049	0
Total Takeaway foods (g)	7030	8515 (21% > Original)	0
**Sugar sweetened drinks**			
Sugar-sweetened soft drink (mL)	13516	18363 (36% > Original)	0
**Artificially sweetened drinks**			
Artificially sweetened soft drink (mL)	2660	1484 (42% < Original)	0
**Items allocated to more than one food group**			
Sandwich, pre-made, white bread, chicken and salad* (g)	469	606	800
Canned meat and vegetable casserole** (g)	1473	1074	0
Orange fruit juice*** (mL)	9143	8405	0

*Divided equally between Grains etc, Lean meats etc, and Vegetables
**Divided equally between Lean meats etc and Vegetables
***Divided equally between Fruit and Discretionary choices – other

Table S2: Composition of the original Healthy Diets ASAP habitual diet pricing tool for mean Australian population and the Low SEG Healthy Diets ASAP habitual diet pricing tool, and recommended diet pricing tool, for a two adults, two children household (Household B)

Food item	Habitual Diet (g/fortnight)		Recommended Diet (g/fortnight) Original & Low SEG Healthy Diets ASAP
	Original Healthy Diets ASAP	Low SEG Healthy Diets ASAP	
**Energy (kJ/day)**	33,602 kJ	32,517 kJ	32,996 kJ
**Water**			
Water, bottled (mL)	5296	3485 (34% < Original)	5296
**Fruit**			
Apples (g)	3497	3638	5460
Bananas (g)	899	795	5460
Oranges (g)	1664	971	5460
Fruit salad, canned in juice (g)	2046	1544	0
Total Fruit (g)	11133	9614 (14% < Original)	16380
**Vegetables and Legumes**			
Potato, loose (g)	1460	1844	2320
Broccoli, loose (g)	422	389	1470
White cabbage, loose (g)	235	175	1470
Iceberg lettuce, whole (g)	795	704	1470
Carrot, loose (g)	753	618	2205
Pumpkin (g)	240	330	2205
Onion, loose (g)	84	106	1638
Tomatoes, loose (g)	488	654	1638
Sweetcorn, canned (g)	206	216	1160
Four bean mix, canned (g)	74	61	1005
Diced tomatoes, canned (g)	235	175	1638
Baked Beans, canned (g)	369	237	1005
Frozen mixed vegetables (g)	1184	746	1638
Frozen peas (g)	273	334	1638
Total Vegetables and Legumes (g)	7584	7136 (6% < Original)	22500
**Grain (Cereal) Foods – Wholegrain and Refined**			
Wholemeal bread, pre-packaged (g)	1054	870	4272
White bread, pre-packaged (g)	3033	3001	893
Rolled oats (g)	870	578	6648
Breakfast cereal, corn flakes (g)	680	509	670
Breakfast cereal, wheat biscuits (g)	430	243	2216
White pasta (g)	1326	988	2042
White rice (g)	1622	1904	2042
Dry wheat crackers, water crackers (g)	258	89	781
Total Grain (Cereal) Foods (g)	9393	8336 (11% < Original)	19564
**Lean Meats and Poultry, Fish, Eggs, Nuts and Seeds**			
Tuna, canned in oil (g)	1052	760	1841
Beef mince, lean (g)	267	163	1168
Lamb loin chops (g)	257	333	1169
Beef rump steak (g)	1056	1042	1172
Eggs (g)	872	884	2208
Chicken, cooked whole (g)	1661	1093	1471
Peanuts, roasted, unsalted (g)	0	0	780
Total Lean Meats and Poultry, Fish, Eggs, Nuts and Seeds (g)	5931	4822 (19% < Original)	9809
**Milk, Yoghurt, Cheese, and Alternatives**			
Cheddar cheese, full fat (g)	624	682	704
Cheddar cheese, reduced fat (g)	44	49	516
Milk, full fat (mL)	5961	7301	6438
Milk, reduced fat (mL)	2929	1839	12000
Flavoured milk (mL)	2416	2187	0
Yoghurt, full fat, plain (g)	204	101	2576
Yoghurt, flavoured reduced fat (g)	676	722	5100
Total Milk, Yoghurt, Cheese, and Alternatives (g)	12854	12881 (0.2% > Original)	27334
**Unsaturated Oils and Spreads (or foods from which these are derived)**			
Sunflower oil (mL)	7	15	291
Olive oil (mL)	7	15	291
Canola margarine (g)	170	197	412
Total Unsaturated Oils and Spreads (g)	184	227 (23% > Original)	994
**Discretionary Choices – other**			
Chicken soup, canned (g)	1340	2219	0
Muffin, commercial (g)	1455	922	0

Food item	Habitual Diet (g/fortnight)		Recommended Diet (g/fortnight) Original & Low SEG Healthy Diets ASAP
	Original Healthy Diets ASAP	Low SEG Healthy Diets ASAP	
Instant noodles, wheat based (g)	381	227	0
White sugar (g)	566	714	0
Cream-filled sweet biscuit, pre-packaged (g)	496	628	0
Muesli bar, pre-packaged (g)	373	339	0
Savoury flavoured biscuits (g)	222	207	0
Nuts, mixed, salted (g)	255	262	0
Confectionary (g)	418	396	0
Chocolate (g)	441	359	0
Potato crisps, pre-packaged (g)	518	650	0
Salad dressing (g)	277	211	0
Tomato sauce (g)	569	511	0
Beef sausages (g)	1047	1036	0
Butter (g)	280	195	0
Ham (g)	189	143	0
Frozen lasagne, pre-packaged (g)	4322	3684	0
Fish fillet crumbed, pre-packaged (g)	302	433	0
Ice cream (g)	1830	1307	0
Total Discretionary Choices – other (g)	18308	17109 (7% < Original)	0
**Alcoholic Drinks**			
Beer, full strength (mL)	4661	5060	0
White wine, sparkling (mL)	863	546	0
Whisky (mL)	266	453	0
Red wine (mL)	1078	519	0
Total Alcoholic Drinks (mL)	6868	6578 (4% < Original)	0
**Takeaway foods**			
Pizza, commercial (g)	1182	1800	0
Meat pie, commercial (g)	1638	1554	0
Hamburger, commercial (g)	2413	2710	0
Potato chips, commercial (g)	670	833	0
Total Takeaway Foods (g)	5903	6897 (17% > Original)	0
**Sugar sweetened beverages**			
Sugar-sweetened soft drink (mL)	12012	16288 (36% > Original)	0
**Artificially sweetened drinks**			
Artificially sweetened soft drink (mL)	2390	1406 (41% < Original)	0
**Items allocated to more than one food group**			
Sandwich, pre-made, white bread, chicken, and salad* (g)	361	462	360
Canned meat and vegetable casserole** (g)	1291	786	0
Orange fruit juice*** (mL)	6053	5331	0

*Divided equally between Grains etc, Lean meats etc, and Vegetables

**Divided equally between Lean meats etc and Vegetables

***Divided equally between Fruit and Discretionary choices - other

Table S3: Composition of the original Healthy Diets ASAP habitual diet pricing tool for mean Australian population and the Low SEG Healthy Diets ASAP habitual diet pricing tool, and recommended diet pricing tool, for an adult female, two children household (Household C)

**Food item**	**Habitual Diet (g/fortnight)**		**Recommended Diet (g/fortnight) Original & Low SEG Healthy Diets ASAP**
	**Original Healthy Diets ASAP**	**Low SEG Healthy Diets ASAP**	
**Energy (kJ/day)**	23,890 kJ	23,424 kJ	23,658 kJ
**Water**			
Water, bottled (mL)	3275	2273 (31% less than Original)	3275
**Fruit**			
Apples (g)	2774	2808	4060
Bananas (g)	606	614	4060
Oranges (g)	1304	560	4060
Fruit salad, canned in juice (g)	1425	1089	0
Total Fruit (g)	8476	7298 (14% less than Original)	12180
**Vegetables and Legumes**			
Potato, loose (g)	944	955	1620
Broccoli, loose (g)	277	209	1120
White cabbage, loose (g)	141	137	1120
Iceberg lettuce, whole (g)	468	378	1120
Carrot, loose (g)	583	485	1680
Pumpkin (g)	154	289	1680
Onion, loose (g)	37	82	1218
Tomatoes, loose (g)	301	397	1218
Sweetcorn, canned (g)	130	179	810
Four bean mix, canned (g)	50	42	480
Diced tomatoes, canned (g)	141	110	1218
Baked Beans, canned (g)	241	96	480
Frozen mixed vegetables (g)	760	482	1218
Frozen peas (g)	167	128	1218
Total Vegetables and Legumes (g)	4884	4269 (13% less than Original)	16200
**Grain (Cereal) Foods – Wholegrain and Refined**			
Wholemeal bread, pre-packaged (g)	683	607	3152
White bread, pre-packaged (g)	2344	2177	669
Rolled oats (g)	615	559	4968
Breakfast cereal, corn flakes (g)	525	289	502
Breakfast cereal, wheat biscuits (g)	263	160	1656
White pasta (g)	942	580	1499
White rice (g)	1098	1248	1499
Dry wheat crackers, water crackers (g)	225	70	585
Total Grain (Cereal) Foods (g)	6753	5837 (14% less than Original)	14530
**Lean Meats and Poultry, Fish, Eggs, Nuts and Seeds**			
Tuna, canned in oil (g)	756	553	1374
Beef mince, lean (g)	157	78	865
Lamb loin chops (g)	83	113	866
Beef rump steak (g)	683	692	868
Eggs (g)	565	524	1648
Chicken, cooked whole (g)	1004	829	1098
Peanuts, roasted, unsalted (g)	0	0	360
Total Lean Meats and Poultry, Fish, Eggs, Nuts and Seeds (g)	3738	3089 (17% less than Original)	7079
**Milk, Yoghurt, Cheese and Alternatives**			
Cheddar cheese, full fat (g)	467	509	544
Cheddar cheese, reduced fat (g)	29	39	396
Milk, full fat (mL)	4702	5328	4938
Milk, reduced fat (mL)	2102	1301	9000
Flavoured milk (mL)	1529	1375	0
Yoghurt, full fat, plain (g)	127	69	1976
Yoghurt, flavoured reduced fat (g)	467	597	3900
Total Milk, Yoghurt, Cheese and Alternatives (g)	9423	9218 (2% less than Original)	20754
**Unsaturated Oils and Spreads (or foods from which these are derived)**			
Sunflower oil (mL)	4	7	160
Olive oil (mL)	4	7	160
Canola margarine (g)	127	149	226
Total Unsaturated Oils and Spreads (g)	135	163 (21% more than Original)	546
**Discretionary Choices – other**			
Chicken soup, canned (g)	715	876	0
Muffin, commercial (g)	1104	666	0

Food item	Habitual Diet (g/fortnight)		Recommended Diet (g/fortnight) Original & Low SEG Healthy Diets ASAP
	Original Healthy Diets ASAP	Low SEG Healthy Diets ASAP	
Instant noodles, wheat based (g)	301	196	0
White sugar (g)	373	430	0
Cream-filled sweet biscuit, pre-packaged (g)	359	441	0
Muesli bar, pre-packaged (g)	301	315	0
Savoury flavoured crackers (g)	182	199	0
Nuts, mixed, salted (g)	154	197	0
Confectionary (g)	373	365	0
Chocolate (g)	333	280	0
Potato crisps, pre-packaged (g)	420	608	0
Salad dressing (g)	187	161	0
Tomato sauce (g)	397	375	0
Beef sausages (g)	701	670	0
Butter (g)	224	109	0
Ham (g)	129	110	0
Frozen lasagne, pre-packaged (g)	3298	2828	0
Fish fillet crumbed, pre-packaged (g)	182	247	0
Ice cream (g)	1561	1077	0
Total Discretionary Choices – other (g)	13661	12377 (9% less than Original)	0
**Alcoholic Drinks**			
Beer, full strength (mL)	428	545	0
White wine, sparkling (mL)	572	433	0
Whisky (mL)	97	371	0
Red wine (mL)	491	218	0
Total Alcoholic Drinks (mL)	1588	1567 (1% less than Original)	0
**Takeaway foods**			
Pizza, commercial (g)	872	1546	0
Meat pie, commercial (g)	1194	1099	0
Hamburger, commercial (g)	1749	1896	0
Potato chips, commercial (g)	446	631	0
Total Takeaway foods (g)	4261	5172 (21% more than Original)	0
**Sugar sweetened drinks**			
Sugar-sweetened soft drink (mL)	8612	13598 (58% more than Original)	0
**Artificially sweetened drinks**			
Artificially sweetened soft drink (mL)	1419	561 (60% less than Original)	0
**Items allocated to more than one food group**			
Sandwich, pre-made, white bread, chicken and salad* (g)	175	442	360
Canned meat and vegetable casserole** (g)	864	305	0
Orange fruit juice*** (mL)	4734	4453	0

*Divided equally between Grains etc, Lean meats etc, and Vegetables

**Divided equally between Lean meats etc and Vegetables

***Divided equally between Fruit and Discretionary choices - other

Table S4: Composition of the original Healthy Diets ASAP habitual diet pricing tool for mean Australian population and the Low SEG Healthy Diets ASAP habitual diet pricing tool, and recommended diet pricing tool for an older retired couple (Household D)

Food item	Habitual Diet (g/fortnight)		Recommended Diet (g/fortnight) Original & Low SEG Healthy Diets ASAP
	Original Healthy Diets ASAP	Low SEG Healthy Diets ASAP	
**Energy (kJ/day)**	14,536 kJ	14,329 kJ	15,095 kJ
**Water**			
Water, bottled (mL)	923	471 (49% less than Original)	923
**Fruit**			
Apples (g)	1271	1027	2800
Bananas (g)	743	680	2800
Oranges (g)	791	645	2800
Fruit salad, canned in juice (g)	2017	1980	0
Total Fruit (g)	5774	5305 (8% less then Original)	8400
**Vegetables and Legumes**			
Potato, loose (g)	1304	1280	800
Broccoli, loose (g)	249	267	700
White cabbage, loose (g)	174	125	700
Iceberg lettuce, whole (g)	418	353	700
Carrot, loose (g)	493	442	1050
Pumpkin (g)	287	287	1050
Onion, loose (g)	128	141	840
Tomatoes, loose (g)	423	461	840
Sweetcorn, canned (g)	161	151	400
Four bean mix, canned (g)	36	13	375
Diced tomatoes, canned (g)	89	98	840
Baked Beans, canned (g)	243	129	375
Frozen mixed vegetables (g)	656	686	840
Frozen peas (g)	234	232	840
Total Vegetables and Legumes (g)	5234	5013 (4% less than Original)	10350
**Grain (Cereal) Foods – Wholegrain and Refined**			
Wholemeal bread, pre-packaged (g)	1055	1057	1280
White bread, pre-packaged (g)	1093	1267	256
Rolled oats (g)	931	1169	2160
Breakfast cereal, corn flakes (g)	331	307	192
Breakfast cereal, wheat biscuits (g)	285	201	720
White pasta (g)	351	410	480
White rice (g)	335	425	480
Dry wheat crackers, water crackers (g)	62	60	224
Total Grain (Cereal) Foods (g)	4471	4925 (10% more than Original)	5792
**Lean Meats and Poultry, Fish, Eggs, Nuts and Seeds**			
Tuna, canned in oil (g)	542	494	934
Beef mince, lean (g)	173	134	433
Lamb loin chops (g)	262	254	433
Beef rump steak (g)	547	443	434
Eggs (g)	515	491	1120
Chicken, cooked whole (g)	801	818	746
Peanuts, roasted, unsalted (g)	0	0	420
Total Lean Meats and Poultry, Fish, Eggs, Nuts and Seeds (g)	3179	2982 (6% less than Original)	4520
**Milk, Yoghurt, Cheese, and Alternatives**			
Cheddar cheese, full fat (g)	250	261	520
Cheddar cheese, reduced fat (g)	25	26	363
Milk, full fat (mL)	2027	1976	4532
Milk, reduced fat (mL)	2427	2071	9126
Flavoured milk (mL)	597	482	0
Yoghurt, full fat, plain (g)	109	114	1813
Yoghurt, flavoured reduced fat (g)	373	350	3650
Total Milk, Yoghurt, Cheese, and Alternatives (g)	5808	5280 (9% less than Original)	20004
**Unsaturated Oils and Spreads (or foods from which these are derived)**			
Sunflower oil (mL)	5	3	132
Olive oil (mL)	5	3	132
Canola margarine (g)	135	140	186
Total Unsaturated Oils and Spreads (g)	145	146 (1% more than Original)	450
**Discretionary Choices – other**			
Chicken soup, canned (g)	1789	1794	0
Muffin, commercial (g)	793	741	0

Food item	Habitual Diet (g/fortnight)		Recommended Diet (g/fortnight) Original & Low SEG Healthy Diets ASAP
	Original Healthy Diets ASAP	Low SEG Healthy Diets ASAP	
Instant noodles, wheat based (g)	65	90	0
White sugar (g)	491	604	0
Cream-filled sweet biscuit, pre-packaged (g)	410	393	0
Muesli bar, pre-packaged (g)	46	40	0
Savoury flavoured crackers (g)	58	47	0
Nuts, mixed, salted (g)	129	127	0
Confectionary (g)	123	124	0
Chocolate (g)	109	126	0
Potato crisps, pre-packaged (g)	52	68	0
Salad dressing (g)	101	71	0
Tomato sauce (g)	216	232	0
Beef sausages (g)	545	521	0
Butter (g)	188	157	0
Ham (g)	103	89	0
Frozen lasagne, pre-packaged (g)	787	875	0
Fish fillet crumbed, pre-packaged (g)	252	264	0
Ice cream (g)	825	852	0
Total Discretionary – other (g)	8034	8188 (2% more than Original)	0
**Alcoholic Drinks**			
Beer, full strength (mL)	2262	2314	0
White wine, sparkling (mL)	907	780	0
Whisky (mL)	166	200	0
Red wine (mL)	968	674	0

Food item	Habitual Diet (g/fortnight)		Recommended Diet (g/fortnight) Original & Low SEG Healthy Diets ASAP
	Original Healthy Diets ASAP	Low SEG Healthy Diets ASAP	
Total Alcoholic Drinks (mL)	4303	3968 (8% less than Original)	0
**Takeaway foods**			
Pizza, commercial (g)	169	71	0
Meat pie, commercial (g)	439	464	0
Hamburger, commercial (g)	352	434	0
Potato chips, commercial (g)	134	184	0
Total Takeaway foods (g)	1094	1153 (5% more than Original)	0
**Sugar sweetened drinks**			
Sugar-sweetened soft drink (mL)	1535	1501 (2% less than Original)	0
**Artificially sweetened drinks**			
Artificially sweetened soft drink (mL)	523	382 (27% less than Original)	0
**Items allocated to more than one food group**			
Sandwich, pre-made, white bread, chicken, and salad* (g)	84	86	320
Canned meat and vegetable casserole** (g)	623	639	0
Orange fruit juice*** (mL)	1903	1945	0

*Divided equally between Grains etc, Lean meats etc, and Vegetables
**Divided equally between Lean meats etc and Vegetables
***Divided equally between Fruit and Discretionary choices - other

Table S5: Composition of the original Healthy Diets ASAP habitual diet pricing tool for mean Australian population and the Low SEG Healthy Diets ASAP habitual diet pricing tool, and recommended diet pricing tool for a single adult male (Household E)

Food item	Habitual Diet (g/fortnight)		Recommended Diet (g/fortnight) Original & Low SEG Healthy Diets ASAP
	Original Healthy Diets ASAP	Low SEG Healthy Diets ASAP	
**Energy (kJ/day)**	9712 kJ	9093 kJ	9338 kJ
**Water**			
Water, bottled (mL)	2021	1211	2021
**Fruit**			
Apples (g)	722	831	1400
Bananas (g)	293	181	1400
Oranges (g)	360	411	1400
Fruit salad, canned in juice (g)	621	454	0
Total Fruit (g)	2656	2317 (13% < Original)	4200
**Vegetables and Legumes**			
Potato, loose (g)	516	889	700
Broccoli, loose (g)	144	180	350
White cabbage, loose (g)	94	38	350
Iceberg lettuce, whole (g)	327	326	350
Carrot, loose (g)	170	134	525
Pumpkin (g)	86	41	525
Onion, loose (g)	48	24	420
Tomatoes, loose (g)	187	257	420
Sweetcorn, canned (g)	77	37	350
Four bean mix, canned (g)	24	19	525
Diced tomatoes, canned (g)	94	65	420
Baked Beans, canned (g)	128	141	525
Frozen mixed vegetables (g)	424	264	420
Frozen peas (g)	106	206	420
Total Vegetables and Legumes (g)	2698	2870 (6% > Original)	6353
**Grain (Cereal) Foods – Wholegrain and Refined**			
Wholemeal bread, pre-packaged (g)	370	263	1120
White bread, pre-packaged (g)	689	824	224
Rolled oats (g)	255	19	1680
Breakfast cereal, corn flakes (g)	155	220	168
Breakfast cereal, wheat biscuits (g)	167	83	560
White pasta (g)	384	408	543
White rice (g)	524	656	543
Dry wheat crackers, water crackers (g)	33	19	196
Total Grain (Cereal) Foods (g)	2640	2499 (5% < Original)	5087
**Lean Meats and Poultry, Fish, Eggs, Nuts and Seeds**			
Tuna, canned in oil (g)	297	207	467
Beef mince, lean (g)	111	85	303
Lamb loin chops (g)	174	220	303
Beef rump steak (g)	373	350	304
Eggs (g)	306	360	560
Chicken, cooked whole (g)	657	264	373
Peanuts, roasted, unsalted (g)	0	0	420
Total Lean Meats and Poultry, Fish, Eggs, Nuts and Seeds (g)	2194	1734 (21% < Original)	2783
**Milk, Yoghurt, Cheese and Alternatives**			
Cheddar cheese, full fat (g)	157	173	160
Cheddar cheese, reduced fat (g)	15	10	120
Milk, full fat (mL)	1259	1973	1500
Milk, reduced fat (mL)	828	539	3000
Flavoured milk (mL)	888	812	0
Yoghurt, full fat, plain (g)	77	32	600
Yoghurt, flavoured reduced fat (g)	209	125	1200
Total Milk, Yoghurt, Cheese and Alternatives (g)	3432	3663 (7% > Original)	6580
**Unsaturated Oils and Spreads (or foods from which these are derived)**			
Sunflower oil (mL)	3	7	131
Olive oil (mL)	3	7	131
Canola margarine (g)	43	48	186
Total Unsaturated Oils and Spreads (g)	49	63 (29% > Original)	448
**Discretionary Choices – other**			
Chicken soup, canned (g)	625	1343	0
Muffin, commercial (g)	352	256	0

Food item	Habitual Diet (g/fortnight)		Recommended Diet (g/fortnight) Original & Low SEG Healthy Diets ASAP
	Original Healthy Diets ASAP	Low SEG Healthy Diets ASAP	
Instant noodles, wheat based (g)	79	31	0
White sugar (g)	194	283	0
Cream-filled sweet biscuit, pre-packaged (g)	136	187	0
Muesli bar, pre-packaged (g)	72	23	0
Savoury flavoured crackers (g)	40	8	0
Nuts, mixed, salted (g)	102	65	0
Confectionary (g)	46	30	0
Chocolate (g)	108	79	0
Potato crisps, pre-packaged (g)	98	42	0
Salad dressing (g)	90	50	0
Tomato sauce (g)	172	136	0
Beef sausages (g)	346	365	0
Butter (g)	56	85	0
Ham (g)	60	33	0
Frozen lasagne, pre-packaged (g)	1024	856	0
Fish fillet crumbed, pre-packaged (g)	120	186	0
Ice cream (g)	269	230	0
Total Discretionary – other (g)	4946	4728 (4% < Original)	0
**Alcoholic Drinks**			
Beer, full strength (mL)	4232	4515	0
White wine, sparkling (mL)	291	114	0
Whisky (mL)	170	82	0
Red wine (mL)	587	301	0
Total Alcoholic Drinks (mL)	5280	5012 (5% < Original)	0
**Takeaway foods**			
Pizza, commercial (g)	310	254	0
Meat pie, commercial (g)	444	456	0
Hamburger, commercial (g)	664	814	0
Potato chips, commercial (g)	225	202	0
Total Takeaway foods (g)	1642	1726 (5% < Original)	0
**Sugar sweetened drinks**			
Sugar-sweetened soft drink (mL)	3400	2690 (21% < Original)	0
**Artificially sweetened drinks0**			
Artificially sweetened soft drink (mL)	972	845 (13% < Original)	0
**Items allocated to more than one food group**			
Sandwich, pre-made, white bread, chicken and salad* (g)	186	20	160
Canned meat and vegetable casserole** (g)	427	481	0
Orange fruit juice*** (mL)	1319	879	0

*Divided equally between Grains etc, Lean meats etc, and Vegetables

**Divided equally between Lean meats etc and Vegetables

***Divided equally between Fruit and Discretionary choices - other

Table S6: Composition of the original Healthy Diets ASAP habitual diet pricing tool for mean Australian population and the Low SEG Healthy Diets ASAP habitual diet pricing tool, and recommended diet pricing tool for an older single female (Household F)

**Food item**	**Habitual Diet (g/fortnight)**		**Recommended Diet (g/fortnight) Original & Low SEG Healthy Diets ASAP**
	**Original Healthy Diets ASAP**	**Low SEG Healthy Diets ASAP**	
**Energy (kJ/day)**	6441 kJ	6136 kJ	7095 kJ
**Water**			
Water, bottled (mL)	434	159 (63% < Original)	434
**Fruit**			
Apples (g)	610	468	1400
Bananas (g)	411	377	1400
Oranges (g)	352	335	1400
Fruit salad, canned in juice (g)	914	897	0
Total Fruit (g)	2767	2607 (6% < Original)	4200
**Vegetables and Legumes**			
Potato, loose (g)	563	562	300
Broccoli, loose (g)	151	150	350
White cabbage, loose (g)	75	60	350
Iceberg lettuce, whole (g)	234	215	350
Carrot, loose (g)	249	217	525
Pumpkin (g)	162	162	525
Onion, loose (g)	38	48	420
Tomatoes, loose (g)	176	187	420
Sweetcorn, canned (g)	97	92	150
Four bean mix, canned (g)	11	6	225
Diced tomatoes, canned (g)	36	46	420
Baked Beans, canned (g)	95	80	225
Frozen mixed vegetables (g)	330	283	420
Frozen peas (g)	127	136	420
Total Vegetables and Legumes (g)	2467	2383 (3% < Original)	5153
**Grain (Cereal) Foods – Wholegrain and Refined**			
Wholemeal bread, pre-packaged (g)	474	477	440
White bread, pre-packaged (g)	433	482	96
Rolled oats (g)	521	601	900
Breakfast cereal, corn flakes (g)	123	109	72
Breakfast cereal, wheat biscuits (g)	110	81	300

**Food item**	**Habitual Diet (g/fortnight)**		**Recommended Diet (g/fortnight) Original & Low SEG Healthy Diets ASAP**
	**Original Healthy Diets ASAP**	**Low SEG Healthy Diets ASAP**	
White pasta (g)	141	159	180
White rice (g)	169	181	180
Dry wheat crackers, water crackers (g)	38	35	84
Total Grain (Cereal) Foods (g)	2015	2134 (6% > Original)	2305
**Lean Meats and Poultry, Fish, Eggs, Nuts and Seeds**			
Tuna, canned in oil (g)	282	257	467
Beef mince, lean (g)	89	71	130
Lamb loin chops (g)	158	169	130
Beef rump steak (g)	252	205	130
Eggs (g)	257	224	560
Chicken, cooked whole (g)	378	388	373
Peanuts, roasted, unsalted (g)	0	0	180
Total Lean Meats and Poultry, Fish, Eggs, Nuts and Seeds (g)	1539	1454 (6% < Original)	2023
**Milk, Yoghurt, Cheese and Alternatives**			
Cheddar cheese, full fat (g)	116	109	280
Cheddar cheese, reduced fat (g)	19	19	198
Milk, full fat (mL)	914	871	2469
Milk, reduced fat (mL)	1234	883	4876
Flavoured milk (mL)	244	235	0
Yoghurt, full fat, plain (g)	63	61	988
Yoghurt, flavoured reduced fat (g)	233	241	1950
Total Milk, Yoghurt, Cheese and Alternatives (g)	2823	2419 (14% < Original)	10761
**Unsaturated Oils and Spreads (or foods from which these are derived)**			
Sunflower oil (mL)	2	2	66
Olive oil (mL)	2	2	66
Canola margarine (g)	60	61	93
Total Unsaturated Oils and Spreads (g)	64	66 (3% > Original)	225
**Discretionary Choices – other**			
Chicken soup, canned (g)	769	859	0
Muffin, commercial (g)	343	299	0
Instant noodles, wheat based (g)	56	70	0

Food item	Habitual Diet (g/fortnight)		Recommended Diet (g/fortnight) Original & Low SEG Healthy Diets ASAP
	Original Healthy Diets ASAP	Low SEG Healthy Diets ASAP	
White sugar (g)	185	208	0
Cream-filled sweet biscuit, pre-packaged (g)	204	190	0
Muesli bar, pre-packaged (g)	19	14	0
Savoury flavoured crackers (g)	20	9	0
Nuts, mixed, salted (g)	54	52	0
Confectionary (g)	56	59	0
Chocolate (g)	49	59	0
Potato crisps, pre-packaged (g)	25	17	0
Salad dressing (g)	65	39	0
Tomato sauce (g)	121	135	0
Beef sausages (g)	217	188	0
Butter (g)	79	47	0
Ham (g)	49	35	0
Frozen lasagne, pre-packaged (g)	389	430	0
Fish fillet crumbed, pre-packaged (g)	119	124	0
Ice cream (g)	307	246	0
Total Discretionary – other (g)	3608	3607 (same as Original)	0
**Alcoholic Drinks**			
Beer, full strength (mL)	244	409	0
White wine, sparkling (mL)	514	423	0
Whisky (mL)	90	119	0
Red wine (mL)	307	268	0
Total Alcoholic Drinks (mL)	1155	1220 (6% > Original)	0
**Takeaway foods**			
Pizza, commercial (g)	64	15	0
Meat pie, commercial (g)	125	178	0
Hamburger, commercial (g)	154	169	0
Potato chips, commercial (g)	62	77	0
Total Takeaway foods (g)	405	440 (9% > Original)	0
**Sugar sweetened drinks**			
Sugar-sweetened soft drink (mL)	617	489 (21% < Original)	0
**Artificially sweetened drinks**			
Artificially sweetened soft drink (mL)	125	75 (40% < Original)	0
**Items allocated to more than one food group**			
Sandwich, pre-made, white bread, chicken and salad* (g)	17	29	160
Canned meat and vegetable casserole** (g)	233	258	0
Orange fruit juice*** (mL)	961	1058	0

*Divided equally between Grains etc, Lean meats etc, and Vegetables

**Divided equally between Lean meats etc and Vegetables

***Divided equally between Fruit and Discretionary choices - other
