# Supplementary material for: Cost and Affordability of Habitual and Recommended Diets in Welfare-Dependent Households in Australia
Source: Nutrients. 2024 Feb 26;16(5):659. doi: 10.3390/nu16050659 (PMC10935407; doi:10.3390/nu16050659)
Supplement: Supplementary file 1 [file nutrients-16-00659-s001.zip › Supplementary Tables S7 to S19 Income calculations and Diet cost details for HH A to F.pdf]

Table S7: Calculations of welfare dependent fortnightly incomes[1] for the six welfare dependent households in April 2023 and September 2023

Household	Two adults, senior female, 3 children (Household A)		Two adults, teen boy, child (Household B)		Adult female, teen boy, child (Household C)		Older, retired couple (Household D)		Single adult male (Household E)		Older single female (Household F)	
	**Apr 2023**	**Sep 2023**	**Apr 2023**	**Sep 2023**	**Apr 2023**	**Sep 2023**	**Apr 2023**	**Sep 2023**	**Apr 2023**	**Sep 2023**	**Apr 2023**	**Sep 2023**
Unemployment Benefit	$631.20 (adult male)	$686.00 (adult male)	$1,262.40 ($631.20 for each adult)	$1,372.00 ($686 for each adult)	$745.20	n/a	n/a	n/a	$693.10	$749.20	n/a	n/a
Parenting Payment (Partnered)	$631.20 (adult female)	$686.00 (adult female)	n/a	n/a	n/a	n/a	n/a	n/a	n/a	n/a	n/a	n/a
Parenting Payment (Single)	n/a	n/a	n/a	n/a	n/a	$972.70	n/a	n/a	n/a	n/a	n/a	n/a
Family Tax Benefit A fortnightly payment	$653.38	$704.20	$455.42	$490.84	$455.42	$490.84	n/a	n/a	n/a	n/a	n/a	n/a
Family Tax Benefit A annual supplement	$94.34 ($817.60 per child, per annum)	$101.50 ($879.65 per child, per annum)	$62.89 ($817.60 per child, per annum)	$67.67 ($879.65 per child, per annum)	$62.89 ($817.60 per child, per annum)	$67.67 ($879.65 per child, per annum)	n/a	n/a	n/a	n/a	n/a	n/a
Family Tax Benefit B fortnightly payment	$87.22	$92.82	$36.40	$37.94	$117.46	$126.56	n/a	n/a	n/a	n/a	n/a	n/a
Family Tax Benefit B annual supplement	$15.30 ($397.85 per family, per annum)	$16.57 ($430.70 per family, per annum)	$15.30 ($397.85 per family, per annum)	$16.57 ($430.70 per family, per annum)	$15.30 ($397.85 per family, per annum)	$16.57 ($430.70 per family, per annum)	n/a	n/a	n/a	n/a	n/a	n/a
Age Pension	$971.50 (senior female)	$1002.50 (senior female)	n/a	n/a	n/a	n/a	$1,464.60 ($732.30 each)	$1,511.40 ($755.70 each)	n/a	n/a	$971.50	$1002.50
Age Pension Supplement	$78.40 (senior female)	$80.10 (senior female)	n/a	n/a	n/a	n/a	$118.20 ($59.10 each)	$120.80 ($60.40 each)	n/a	n/a	$78.40	$80.10
Clean Energy Supplement	$29.90	$29.90	$15.80	$15.80	$9.50	$9.50	$21.20	$21.20	$8.80	$8.80	$14.10	$14.10
Rent Assistance	n/a (public housing)	n/a (public housing)	$184.94	$217.28	$184.94	$217.28	$148.00	$174.00	$83.70	$117.45	$157.20	$184.80
**Fortnightly income Total**	**$3,192.44**	**$3,399.58**	**$2,033.15**	**$2,218.09**	**$1,590.71**	**$1,901.11**	**$1,752.00**	**$1,827.40**	**$785.60**	**$875.45**	**$1,221.20**	**$1,281.50**

n/a = not applicable 1. Services Australia. Payment and Service Finder 2023 Available from: https://www.servicesaustralia.gov.au/

Table S8: Diet cost and affordability by food group and food group components of habitual and recommended diets, for a two adult, senior female, three children household (Household A) in Greater Brisbane, April 2023

**Mean food group and food group component costs per fortnight for Greater Brisbane**																		
**Food group and food group component**	**Broader population household**						**Welfare dependent household**											
	**Habitual Diet – branded products**			**Recommended Diet – branded products**			**Habitual Diet – branded products**			**Recommended Diet – branded products**			**Habitual Diet – cheapest products**			**Recommended Diet – cheapest products**		
	**Cost (A$)**	**Std Error (A$)**	**Proportion of total diet cost (%)**	**Cost (A$)**	**Std Error (A$)**	**Proportion of total diet cost (%)**	**Cost (A$)**	**Std Error (A$)**	**Proportion of total diet cost (%)**	**Cost (A$)**	**Std Error (A$)**	**Proportion of total diet cost (%)**	**Cost (A$)**	**Std Error (A$)**	**Proportion of total diet cost (%)**	**Cost (A$)**	**Std Error (A$)**	**Proportion of total diet cost (%)**
Water	$23.83	$0.59	1.9%	$23.83	$0.59	2.2%	$15.80	$0.39	1.3%	$23.83	$0.59	2.2%	$5.48	<$0.01	0.7%	$8.26	<$0.01	1.1%
Fruit	$113.90	$2.04	9.1%	$145.38	$5.92	13.7%	$96.76	$1.63	7.9%	$145.38	$5.92	13.7%	$74.02	$0.41	9.2%	$139.55	$1.91	19.0%
Veg. & Legumes	$74.22	$0.91	5.9%	$181.21	$2.60	17.1%	$68.84	$0.75	5.6%	$181.21	$2.60	17.1%	$55.55	$0.28	6.9%	$138.98	$0.71	18.9%
Grains & Cereals	$80.27	$1.22	6.4%	$173.61	$2.83	16.3%	$72.61	$1.15	5.9%	$173.61	$2.83	16.3%	$35.82	$0.21	4.5%	$72.84	$0.21	9.9%
Meats, nuts, seeds, egg & alternatives	$165.54	$1.90	13.3%	$304.07	$3.23	28.6%	$147.41	$1.67	12.0%	$304.07	$3.23	28.6%	$117.06	$0.55	14.6%	$236.46	$1.02	32.2%
Milk, yoghurt, cheese & alternatives	$101.35	$0.88	8.1%	$218.21	$1.99	20.5%	$92.95	$0.77	7.6%	$218.21	$1.99	20.5%	$61.41	$0.33	7.7%	$130.16	$0.92	17.7%
Unsaturated oils & spreads	$3.03	$0.05	0.2%	$16.41	$0.22	1.5%	$3.90	$0.06	0.3%	$16.41	$0.22	1.5%	$1.80	$0.04	0.2%	$8.47	$0.06	1.2%
Artificially sweetened soft drink	$7.67	$0.09	0.6%	--	--	--	$4.28	$0.05	0.3%	--	--	--	$1.29	<$0.01	0.2%	--	--	--
Discretionary choices - other	$312.46	$2.47	25.0%	--	--	--	$294.77	$2.23	24.1%	--	--	--	$166.73	$0.36	20.8%	--	--	--
Alcoholic drinks	$114.44	$0.62	9.2%	--	--	--	$114.80	$0.66	9.4%	--	--	--	$85.41	$1.53	10.7%	--	--	--
Take-away foods	$212.46	$1.60	17.0%	--	--	--	$259.98	$1.75	21.2%	--	--	--	$180.10	$2.09	22.5%	--	--	--
Sugar sweetened soft drinks	$39.07	$0.45	3.1%	--	--	--	$53.09	$0.61	4.3%	--	--	--	$15.91	<$0.01	2.0%	--	--	--
**Total cost**	**$1,248.26**	**$8.56**	**100%**	**$1,062.72**	**$13.93**	**100%**	**$1,225.19**	**$7.80**	**100%**	**$1,062.72**	**$13.93**	**100%**	**$800.57**	**$2.62**	**100%**	**$734.72**	**$2.42**	**100%**
Healthy foods and drinks	$569.82	$6.10	45.6%	$1,062.72	$13.92	100%	$502.55	$5.07	41.0%	$1,062.72	$13.92	100%	$352.42	$0.86	44.0%	$734.72	$2.42	100%
Discretionary foods and drinks	$678.44	$3.39	54.4%	--	--	--	$722.64	$3.52	59.0%	--	--	--	$448.15	$2.45	56.0%	--	--	--

**Affordability of diets**												
**Income amount (A$)**	**Broader population household**				**Welfare dependent household**							
	**Habitual Diet – branded products**		**Recommended Diet – branded products**		**Habitual Diet – branded products**		**Recommended Diet – branded products**		**Habitual Diet – cheapest products**		**Recommended Diet – cheapest products**	
	**Affordability**	**Std error**	**Affordability**	**Std error**	**Affordability**	**Std error**	**Affordability**	**Std error**	**Affordability**	**Std error**	**Affordability**	**Std error**
April 2023 Welfare only household income $3,192 per fortnight	39.10%	0.27%	33.29%	0.44%	38.38%	0.25%	33.29%	0.44%	25.08%	0.08%	23.01%	0.08%

Table S9: Diet cost and affordability by food group and food group components of habitual and recommended diets, for a two adult, two children household (Household B) in Greater Brisbane, April 2023

**Mean food group and food group component costs per fortnight for Greater Brisbane**																		
**Food group and food group component**	**Broader population household**						**Welfare dependent household**											
	**Habitual Diet – branded products**			**Recommended Diet – branded products**			**Habitual Diet – branded products**			**Recommended Diet – branded products**			**Habitual Diet – cheapest products**			**Recommended Diet – cheapest products**		
	**Cost (A$)**	**Std Error (A$)**	**Proportion of total diet cost (%)**	**Cost (A$)**	**Std Error (A$)**	**Proportion of total diet cost (%)**	**Cost (A$)**	**Std Error (A$)**	**Proportion of total diet cost (%)**	**Cost (A$)**	**Std Error (A$)**	**Proportion of total diet cost (%)**	**Cost (A$)**	**Std Error (A$)**	**Proportion of total diet cost (%)**	**Cost (A$)**	**Std Error (A$)**	**Proportion of total diet cost (%)**
Water	$21.22	$0.52	2.3%	$21.22	$0.52	2.8%	$13.96	$0.34	1.5%	$21.22	$0.52	2.8%	$4.84	<$0.01	0.8%	$7.36	<$0.00	1.4%
Fruit	$69.91	$1.33	7.6%	$100.35	$4.08	13.3%	$58.43	$1.02	6.5%	$100.35	$4.08	13.3%	$44.99	$0.26	7.6%	$96.32	$1.32	18.5%
Veg. & Legumes	$51.67	$0.63	5.6%	$126.68	$1.78	16.7%	$47.43	$0.51	5.3%	$126.68	$1.78	16.7%	$38.59	$0.19	6.5%	$96.91	$0.49	18.6%
Grains & Cereals	$54.76	$0.80	6.0%	$133.63	$2.18	17.7%	$46.53	$0.72	5.2%	$133.63	$2.18	17.7%	$23.84	$0.15	4.0%	$55.73	$0.17	10.7%
Meats, nuts, seeds, egg & alternatives	$118.04	$1.40	12.9%	$223.38	$2.36	29.5%	$104.61	$1.22	11.6%	$223.38	$2.36	29.5%	$83.64	$0.40	14.1%	$176.12	$0.76	33.7%
Milk, yoghurt, cheese & alternatives	$65.85	$0.57	7.2%	$138.97	$1.26	18.4%	$66.05	$0.56	7.3%	$138.97	$1.26	18.4%	$44.00	$0.25	7.4%	$82.96	$0.58	15.9%
Unsaturated oils & spreads	$1.97	$0.03	0.2%	$12.56	$0.17	1.7%	$2.48	$0.04	0.3%	$12.56	$0.17	1.7%	$1.15	$0.03	0.2%	$6.49	$0.05	1.2%
Artificially sweetened soft drink	$6.89	$0.08	0.8%	--	--	--	$4.05	$0.05	0.4%	--	--	--	$1.22	<$0.01	0.2%	--	--	--
Discretionary choices - other	$220.56	$1.90	24.0%	--	--	--	$208.66	$1.71	23.1%	--	--	--	$116.99	$0.23	19.8%	--	--	--
Alcoholic drinks	$93.59	$0.49	10.2%	--	--	--	$92.56	$0.54	10.3%	--	--	--	$70.66	$1.45	12.0%	--	--	--
Take-away foods	$178.95	$1.33	19.5%	--	--	--	$211.04	$1.40	23.4%	--	--	--	$147.22	$1.67	24.9%	--	--	--
Sugar sweetened soft drinks	$34.73	$0.40	3.8%	--	--	--	$47.09	$0.54	5.2%	--	--	--	$14.12	<$0.01	2.4%	--	--	--
**Total cost**	**$918.14**	**$6.14**	**100%**	**$756.80**	**$9.87**	**100%**	**$902.90**	**$5.59**	**100%**	**$756.80**	**$9.87**	**100%**	**$591.26**	**$2.18**	**100%**	**$521.88**	**$1.69**	**100%**
Healthy foods and drinks	$390.31	$4.24	42.5%	$756.80	$9.87	100%	$343.55	$3.46	38.0%	$756.80	$9.87	100%	$242.26	$0.61	41.0%	$521.88	$1.69	100%
Discretionary foods and drinks	$527.82	$2.69	57.5%	--	--	--	$559.35	$2.77	62.0%	--	--	--	$349.00	$2.09	59.0%	--	--	--

**Affordability of diets**												
**Income amount (A$)**	**Broader population household**				**Welfare dependent household**							
	**Habitual Diet – branded products**		**Recommended Diet – branded products**		**Habitual Diet – branded products**		**Recommended Diet – branded products**		**Habitual Diet – cheapest products**		**Recommended Diet – cheapest products**	
	**Affordability**	**Std error**	**Affordability**	**Std error**	**Affordability**	**Std error**	**Affordability**	**Std error**	**Affordability**	**Std error**	**Affordability**	**Std error**
April 2023 Welfare only household income $2,033 per fortnight	45.16%	0.30%	37.22%	0.49%	44.41%	0.27%	37.22%	0.49%	29.08%	0.11%	25.67%	0.08%

Table S10: Diet cost and affordability by food group and food group components of habitual and recommended diets, for an adult female, two children household (Household C) in Greater Brisbane, April 2023

**Mean food group and food group component costs per fortnight for Greater Brisbane**																		
**Food group and food group component**	**Broader population household**						**Welfare dependent household**											
	**Habitual Diet – branded products**			**Recommended Diet – branded products**			**Habitual Diet – branded products**			**Recommended Diet – branded products**			**Habitual Diet – cheapest products**			**Recommended Diet – cheapest products**		
	**Cost (A$)**	**Std Error (A$)**	**Proportion of total diet cost (%)**	**Cost (A$)**	**Std Error (A$)**	**Proportion of total diet cost (%)**	**Cost (A$)**	**Std Error (A$)**	**Proportion of total diet cost (%)**	**Cost (A$)**	**Std Error (A$)**	**Proportion of total diet cost (%)**	**Cost (A$)**	**Std Error (A$)**	**Proportion of total diet cost (%)**	**Cost (A$)**	**Std Error (A$)**	**Proportion of total diet cost (%)**
Water	$13.12	$0.32	2.2%	$13.12	$0.32	2.4%	$9.11	$0.22	1.5%	$13.12	$0.32	2.4%	$3.16	<$0.01	0.8%	$4.55	<$0.01	1.2%
Fruit	$52.58	$1.02	8.6%	$74.62	$3.04	13.4%	$43.33	$0.70	7.0%	$74.62	$3.04	13.4%	$32.90	$0.17	8.3%	$71.63	$0.98	18.7%
Veg. & Legumes	$32.42	$0.39	5.3%	$90.92	$1.33	16.4%	$29.05	$0.29	4.7%	$90.92	$1.33	16.4%	$24.27	$0.12	6.1%	$70.64	$0.36	18.4%
Grains & Cereals	$39.05	$0.59	6.4%	$99.33	$1.63	17.9%	$34.64	$0.52	5.6%	$99.33	$1.63	17.9%	$18.36	$0.10	4.7%	$41.26	$0.12	10.7%
Meats, nuts, seeds, egg & alternatives	$73.59	$0.92	12.1%	$164.12	$1.75	29.6%	$65.81	$0.79	10.6%	$164.12	$1.75	29.6%	$51.68	$0.25	13.1%	$128.98	$0.57	33.6%
Milk, yoghurt, cheese & alternatives	$46.84	$0.39	7.7%	$106.18	$0.96	19.1%	$47.42	$0.39	7.6%	$106.18	$0.96	19.1%	$31.28	$0.17	7.9%	$63.35	$0.45	16.5%
Unsaturated oils & spreads	$1.44	$0.02	0.2%	$6.90	$0.09	1.2%	$1.75	$0.03	0.3%	$6.90	$0.09	1.2%	$0.80	$0.02	0.2%	$3.56	$0.03	0.9%
Artificially sweetened soft drink	$4.09	$0.05	0.7%	--	--	--	$1.62	$0.02	0.3%	--	--	--	$0.49	<$0.01	0.1%	--	--	--
Discretionary choices - other	$164.29	$1.42	27.0%	--	--	--	$154.74	$1.27	24.8%	--	--	--	$83.89	$0.16	21.2%	--	--	--
Alcoholic drinks	$27.78	$0.19	4.6%	--	--	--	$38.13	$0.42	6.1%	--	--	--	$29.43	$0.19	7.5%	--	--	--
Take-away foods	$128.94	$0.95	21.2%	--	--	--	$157.88	$1.02	25.4%	--	--	--	$106.76	$1.24	27.0%	--	--	--
Sugar sweetened soft drinks	$24.90	$0.29	4.1%	--	--	--	$39.31	$0.45	6.3%	--	--	--	$11.78	<$0.01	3.0%	--	--	--
**Total cost**	**$609.05**	**$4.31**	**100%**	**$555.19**	**$7.26**	**100%**	**$622.79**	**$3.96**	**100%**	**$555.19**	**$7.26**	**100%**	**$394.81**	**$1.24**	**100%**	**$383.97**	**$1.25**	**100%**
Healthy foods and drinks	$263.14	$2.92	43.2%	$555.19	$7.26	100%	$232.72	$2.29	37.4%	$555.19	$7.26	100%	$162.93	$0.37	41.3%	$383.97	$1.25	100%
Discretionary foods and drinks	$345.91	$1.92	56.8%	--	--	--	$390.06	$2.10	62.6%	--	--	--	$231.87	$1.31	58.7%	--	--	--

**Affordability of diets**												
**Income amount (A$)**	**Broader population household**				**Welfare dependent household**							
	**Habitual Diet – branded products**		**Recommended Diet – branded products**		**Habitual Diet – branded products**		**Recommended Diet – branded products**		**Habitual Diet – cheapest products**		**Recommended Diet – cheapest products**	
	**Affordability**	**Std error**	**Affordability**	**Std error**	**Affordability**	**Std error**	**Affordability**	**Std error**	**Affordability**	**Std error**	**Affordability**	**Std error**
April 2023 Welfare only household income $1,591 per fortnight	38.29%	0.27%	34.90%	0.46%	39.15%	0.25%	34.90%	0.46%	24.82%	0.08%	24.14%	0.08%

Table S11: Diet cost and affordability by food group and food group components of habitual and recommended diets, for an older retired couple household (Household D) in Greater Brisbane, April 2023

Mean food group and food group component costs per fortnight for Greater Brisbane																		
**Food group and food group component**	**Broader population household**						**Welfare dependent household**											
	**Habitual Diet – branded products**			**Recommended Diet – branded products**			**Habitual Diet – branded products**			**Recommended Diet – branded products**			**Habitual Diet – cheapest products**			**Recommended Diet – cheapest products**		
	**Cost (A$)**	**Std Error (A$)**	**Proportion of total diet cost (%)**	**Cost (A$)**	**Std Error (A$)**	**Proportion of total diet cost (%)**	**Cost (A$)**	**Std Error (A$)**	**Proportion of total diet cost (%)**	**Cost (A$)**	**Std Error (A$)**	**Proportion of total diet cost (%)**	**Cost (A$)**	**Std Error (A$)**	**Proportion of total diet cost (%)**	**Cost (A$)**	**Std Error (A$)**	**Proportion of total diet cost (%)**
Water	$3.70	$0.09	1.0%	$3.70	$0.09	1.0%	$1.89	$0.05	0.5%	$3.70	$0.09	1.0%	$0.65	<$0.01	0.3%	$1.28	<$0.01	0.5%
Fruit	$41.60	$0.67	10.7%	$51.46	$2.09	14.2%	$38.52	$0.57	10.3%	$51.46	$2.09	14.2%	$28.73	$0.18	11.5%	$49.40	$0.67	19.5%
Veg. & Legumes	$31.61	$0.38	8.2%	$59.66	$0.87	16.5%	$30.56	$0.38	8.2%	$59.66	$0.87	16.5%	$24.05	$0.15	9.6%	$46.06	$0.24	18.2%
Grains & Cereals	$28.37	$0.49	7.3%	$42.98	$0.69	11.9%	$30.38	$0.56	8.2%	$42.98	$0.69	11.9%	$13.07	$0.07	5.2%	$18.94	$0.05	7.5%
Meats, nuts, seeds, egg & alternatives	$66.48	$0.77	17.2%	$97.39	$1.04	26.9%	$60.11	$0.69	16.1%	$97.39	$1.04	26.9%	$46.70	$0.21	18.7%	$74.23	$0.31	29.3%
Milk, yoghurt, cheese & alternatives	$26.82	$0.22	6.9%	$100.60	$0.91	27.8%	$25.28	$0.21	6.8%	$100.60	$0.91	27.8%	$16.41	$0.08	6.6%	$60.16	$0.42	23.8%
Unsaturated oils & spreads	$1.55	$0.03	0.4%	$5.69	$0.08	1.6%	$1.55	$0.03	0.4%	$5.69	$0.08	1.6%	$0.70	$0.02	0.3%	$2.94	$0.02	1.2%
Artificially sweetened soft drink	$1.51	$0.02	0.4%	--	--	--	$1.10	$0.01	0.3%	--	--	--	$0.33	<$0.01	0.1%	--	--	--
Discretionary choices - other	$85.77	$0.54	22.2%	--	--	--	$86.39	$0.55	23.2%	--	--	--	$54.09	$0.18	21.6%	--	--	--
Alcoholic drinks	$63.52	$0.40	16.4%	--	--	--	$58.01	$0.32	15.6%	--	--	--	$38.94	$0.61	15.6%	--	--	--
Take-away foods	$31.74	$0.33	8.2%	--	--	--	$34.06	$0.37	9.2%	--	--	--	$25.07	$0.38	10.0%	--	--	--
Sugar sweetened soft drinks	$4.44	$0.05	1.1%	--	--	--	$4.34	$0.05	1.2%	--	--	--	$1.30	<$0.01	0.5%	--	--	--
**Total cost**	**$387.11**	**$2.50**	**100%**	**$361.48**	**$4.64**	**100%**	**$372.19**	**$2.45**	**100%**	**$361.48**	**$4.64**	**100%**	**$250.03**	**$0.84**	**100%**	**$253.00**	**$0.84**	**100%**
Healthy foods and drinks	$201.64	$2.03	52.1%	$361.48	$4.64	100%	$189.39	$1.88	50.9%	$361.48	$4.64	100%	$130.64	$0.36	52.2%	$253.00	$0.84	100%
Discretionary foods and drinks	$185.47	$0.70	47.9%	--	--	--	$182.80	$0.75	49.1%	--	--	--	$119.40	$0.68	47.8%	--	--	--

Affordability of diets												
**Income amount (A$)**	**Broader population household**				**Welfare dependent household**							
	**Habitual Diet – branded products**		**Recommended Diet – branded products**		**Habitual Diet – branded products**		**Recommended Diet – branded products**		**Habitual Diet – cheapest products**		**Recommended Diet – cheapest products**	
	**Affordability**	**Std error**	**Affordability**	**Std error**	**Affordability**	**Std error**	**Affordability**	**Std error**	**Affordability**	**Std error**	**Affordability**	**Std error**
April 2023 Welfare only household income $1,752 per fortnight	22.10%	0.14%	20.63%	0.26%	21.24%	0.14%	20.63%	0.26%	14.27%	0.05%	14.44%	0.05%

Table S12: Diet cost and affordability by food group and food group components of habitual and recommended diets, for a single adult male household (Household E) in Greater Brisbane, April 2023

Mean food group and food group component costs per fortnight for Greater Brisbane

Food group and food group component	Broader population household						Welfare dependent household											
	Habitual Diet – branded products			Recommended Diet – branded products			Habitual Diet – branded products			Recommended Diet – branded products			Habitual Diet – cheapest products			Recommended Diet – cheapest products		
	Cost (A$)	Std Error (A$)	Proportion of total diet cost (%)	Cost (A$)	Std Error (A$)	Proportion of total diet cost (%)	Cost (A$)	Std Error (A$)	Proportion of total diet cost (%)	Cost (A$)	Std Error (A$)	Proportion of total diet cost (%)	Cost (A$)	Std Error (A$)	Proportion of total diet cost (%)	Cost (A$)	Std Error (A$)	Proportion of total diet cost (%)
Water	$8.10	$0.20	2.6%	$8.10	$0.20	4.0%	$4.85	$0.12	0.9%	$8.10	$0.20	4.0%	$1.68	<$0.01	0.86%	$2.81	<$0.01	2.04%
Fruit	$17.33	$0.30	5.6%	$25.73	$1.05	12.8%	$15.10	$0.32	6.2%	$25.73	$1.05	12.8%	$12.09	$0.10	6.15%	$24.70	$0.34	17.91%
Veg. & Legumes	$19.27	$0.23	6.2%	$35.76	$0.46	17.7%	$18.41	$0.21	7.3%	$35.76	$0.46	17.7%	$14.32	$0.08	7.29%	$26.27	$0.12	19.05%
Grains & Cereals	$15.72	$0.21	5.1%	$34.30	$0.55	17.0%	$11.92	$0.19	2.8%	$34.30	$0.55	17.0%	$5.48	$0.05	2.79%	$14.47	$0.04	10.49%
Meats, nuts, seeds, egg & alternatives	$44.56	$0.48	14.4%	$59.25	$0.62	29.4%	$38.86	$0.44	16.3%	$59.25	$0.62	29.4%	$31.96	$0.15	16.27%	$47.13	$0.19	34.17%
Milk, yoghurt, cheese & alternatives	$19.02	$0.18	6.1%	$32.79	$0.30	16.3%	$18.64	$0.17	6.5%	$32.79	$0.30	16.3%	$12.72	$0.08	6.48%	$19.62	$0.14	14.22%
Unsaturated oils & spreads	$0.53	$0.01	0.2%	$5.66	$0.08	2.8%	$0.70	$0.01	0.2%	$5.66	$0.08	2.8%	$0.33	$0.01	0.17%	$2.92	$0.02	2.12%
Artificially sweetened soft drink	$2.80	$0.03	0.9%	--	--	--	$2.44	$0.03	0.4%	--	--	--	$0.73	<$0.01	0.37%	--	--	--
Discretionary choices - other	$56.29	$0.48	18.2%	--	--	--	$53.84	$0.44	16.8%	--	--	--	$33.06	$0.11	16.83%	--	--	--
Alcoholic drinks	$65.86	$0.31	21.3%	--	--	--	$54.45	$0.25	21.0%	--	--	--	$41.24	$1.33	21.00%	--	--	--
Take-away foods	$50.05	$0.39	16.2%	--	--	--	$53.18	$0.38	20.6%	--	--	--	$40.48	$0.42	20.61%	--	--	--
Sugar sweetened soft drinks	$9.83	$0.11	3.2%	--	--	--	$7.78	$0.09	1.2%	--	--	--	$2.33	<$0.01	1.19%	--	--	--
**Total cost**	**$309.35**	**$1.85**	**100%**	**$201.60**	**$2.62**	**100%**	**$280.15**	**$1.63**	**100%**	**$201.60**	**$2.62**	**100%**	**$196.41**	**$1.49**	**100%**	**$137.91**	**$0.44**	**100%**
Healthy foods and drinks	$127.33	$1.29	41.2%	$201.60	$2.62	100%	$110.90	$1.14	39.6%	$201.60	$2.62	100%	$79.31	$0.25	40.38%	$137.91	$0.44	100%
Discretionary foods and drinks	$182.02	$0.80	58.8%	--	--	--	$169.24	$0.69	60.4%	--	--	--	$117.11	$1.38	59.62%	--	--	--

Affordability of diets

Income amount (A$)	Broader population household				Welfare dependent household							
	Habitual Diet – branded products		Recommended Diet – branded products		Habitual Diet – branded products		Recommended Diet – branded products		Habitual Diet – cheapest products		Recommended Diet – cheapest products	
	Affordability	Std error	Affordability	Std error	Affordability	Std error	Affordability	Std error	Affordability	Std error	Affordability	Std error
April 2023 Welfare only household income $786 per fortnight	39.38%	0.23%	25.66%	0.33%	35.66%	0.21%	25.66%	0.33%	25.00%	0.19%	17.56%	0.06%

Table S13: Diet cost and affordability by food group and food group components of habitual and recommended diets, for an older single female household (Household F) in Greater Brisbane, April 2023

**Mean food group and food group component costs per fortnight for Greater Brisbane**																		
**Food group and food group component**	**Broader population household**						**Welfare dependent household**											
	**Habitual Diet – branded products**			**Recommended Diet – branded products**			**Habitual Diet – branded products**			**Recommended Diet – branded products**			**Habitual Diet – cheapest products**			**Recommended Diet – cheapest products**		
	**Cost (A$)**	**Std Error (A$)**	**Proportion of total diet cost (%)**	**Cost (A$)**	**Std Error (A$)**	**Proportion of total diet cost (%)**	**Cost (A$)**	**Std Error (A$)**	**Proportion of total diet cost (%)**	**Cost (A$)**	**Std Error (A$)**	**Proportion of total diet cost (%)**	**Cost (A$)**	**Std Error (A$)**	**Proportion of total diet cost (%)**	**Cost (A$)**	**Std Error (A$)**	**Proportion of total diet cost (%)**
Water	$1.74	$0.04	1.0%	$1.74	$0.04	1.0%	$0.64	$0.02	0.4%	$1.74	$0.04	1.0%	$0.22	<$0.01	0.2%	$0.60	<$0.01	0.5%
Fruit	$19.59	$0.32	11.5%	$25.73	$1.05	15.1%	$18.51	$0.29	11.2%	$25.73	$1.05	15.1%	$13.87	$0.09	12.5%	$24.70	$0.34	21.0%
Veg. & Legumes	$14.68	$0.20	8.6%	$29.13	$0.43	17.1%	$14.39	$0.19	8.7%	$29.13	$0.43	17.1%	$11.22	$0.06	10.1%	$22.52	$0.12	19.1%
Grains & Cereals	$12.65	$0.23	7.4%	$17.37	$0.28	10.2%	$13.29	$0.25	8.0%	$17.37	$0.28	10.2%	$5.53	$0.03	5.0%	$7.80	$0.02	6.6%
Meats, nuts, seeds, egg & alternatives	$33.22	$0.39	19.5%	$39.49	$0.44	23.2%	$30.93	$0.36	18.6%	$39.49	$0.44	23.2%	$24.08	$0.12	21.6%	$28.37	$0.12	24.1%
Milk, yoghurt, cheese & alternatives	$13.15	$0.11	7.7%	$54.18	$0.49	31.8%	$12.05	$0.10	7.3%	$54.18	$0.49	31.8%	$7.73	$0.04	6.9%	$32.42	$0.23	27.5%
Unsaturated oils & spreads	$0.68	$0.01	0.4%	$2.84	$0.04	1.7%	$0.69	$0.01	0.4%	$2.84	$0.04	1.7%	$0.31	$0.01	0.3%	$1.47	$0.01	1.2%
Artificially sweetened soft drink	$0.36	<$0.00	0.2%	--	--	--	$0.22	$0.00	0.1%	--	--	--	$0.07	<$0.01	0.1%	--	--	--
Discretionary choices - other	$39.22	$0.25	23.1%	--	--	--	$38.51	$0.25	23.2%	--	--	--	$23.51	$0.08	21.1%	--	--	--
Alcoholic drinks	$20.86	$0.14	12.3%	--	--	--	$22.21	$0.13	13.4%	--	--	--	$14.73	$0.10	13.2%	--	--	--
Take-away foods	$12.21	$0.11	7.2%	--	--	--	$13.02	$0.14	7.9%	--	--	--	$9.63	$0.14	8.6%	--	--	--
Sugar sweetened soft drinks	$1.78	$0.02	1.0%	--	--	--	$1.41	$0.02	0.9%	--	--	--	$0.42	<$0.01	0.4%	--	--	--
**Total cost**	**$170.15**	**$1.20**	**100%**	**$170.48**	**$2.23**	**100%**	**$165.86**	**$1.17**	**100%**	**$170.48**	**$2.23**	**100%**	**$111.32**	**$0.24**	**100%**	**$117.88**	**$0.41**	**100%**
Healthy foods and drinks	$96.07	$1.00	56.5%	$170.48	$2.23	100%	$90.71	$0.92	54.7%	$170.48	$2.23	100%	$63.02	$0.18	56.6%	$117.88	$0.41	100%
Discretionary foods and drinks	$74.07	$0.30	43.5%	--	--	--	$75.15	$0.32	45.3%	--	--	--	$48.30	$0.16	43.4%	--	--	--

**Affordability of diets**												
**Income amount (A$)**	**Broader population household**				**Welfare dependent household**							
	**Habitual Diet – branded products**		**Recommended Diet – branded products**		**Habitual Diet – branded products**		**Recommended Diet – branded products**		**Habitual Diet – cheapest products**		**Recommended Diet – cheapest products**	
	**Affordability**	**Std error**	**Affordability**	**Std error**	**Affordability**	**Std error**	**Affordability**	**Std error**	**Affordability**	**Std error**	**Affordability**	**Std error**
April 2023 Welfare only household income $1,221 per fortnight	13.93%	0.10%	13.96%	0.18%	13.58%	0.10%	13.96%	0.18%	9.12%	0.02%	9.65%	0.03%

Table S14: Diet cost and affordability by food group and food group components of habitual and recommended diets, for a two adult, senior female, three children household (Household A) in Greater Brisbane, September 2023

**Mean food group and food group component costs per fortnight for Greater Brisbane**																		
**Food group and food group component**	**Broader population household**						**Welfare dependent household**											
	**Habitual Diet – branded products**			**Recommended Diet – branded products**			**Habitual Diet – branded products**			**Recommended Diet – branded products**			**Habitual Diet – cheapest products**			**Recommended Diet – cheapest products**		
	**Cost (A$)**	**Std Error (A$)**	**Proportion of total diet cost (%)**	**Cost (A$)**	**Std Error (A$)**	**Proportion of total diet cost (%)**	**Cost (A$)**	**Std Error (A$)**	**Proportion of total diet cost (%)**	**Cost (A$)**	**Std Error (A$)**	**Proportion of total diet cost (%)**	**Cost (A$)**	**Std Error (A$)**	**Proportion of total diet cost (%)**	**Cost (A$)**	**Std Error (A$)**	**Proportion of total diet cost (%)**
Water	$24.57	$0.61	1.9%	$24.57	$0.61	2.3%	$16.29	$0.40	1.3%	$24.57	$0.61	2.3%	$5.65	<$0.01	0.7%	$8.52	<$0.01	1.1%
Fruit	$113.12	$2.01	9.0%	$143.34	$5.83	13.3%	$96.16	$1.61	7.7%	$143.34	$5.83	13.3%	$73.33	$0.40	9.1%	$137.59	$1.88	18.5%
Veg. & Legumes	$76.44	$0.95	6.1%	$188.11	$2.70	17.4%	$71.04	$0.78	5.7%	$188.11	$2.70	17.4%	$57.32	$0.30	7.1%	$144.23	$0.73	19.4%
Grains & Cereals	$82.50	$1.26	6.5%	$177.29	$2.89	16.4%	$74.77	$1.20	6.0%	$177.29	$2.89	16.4%	$36.95	$0.22	4.6%	$74.58	$0.22	10.0%
Meats, nuts, seeds, egg & alternatives	$165.84	$1.90	13.1%	$305.34	$3.25	28.3%	$147.67	$1.67	11.9%	$305.34	$3.25	28.3%	$117.04	$0.55	14.5%	$236.93	$1.03	31.9%
Milk, yoghurt, cheese & alternatives	$103.05	$0.90	8.2%	$223.27	$2.06	20.7%	$94.37	$0.79	7.6%	$223.27	$2.06	20.7%	$62.30	$0.33	7.7%	$132.79	$0.95	17.9%
Unsaturated oils & spreads	$3.04	$0.05	0.2%	$16.50	$0.22	1.5%	$3.91	$0.06	0.3%	$16.50	$0.22	1.5%	$1.81	$0.04	0.2%	$8.52	$0.06	1.1%
Artificially sweetened soft drink	$7.91	$0.09	0.6%	--	--	--	$4.41	$0.05	0.4%	--	--	--	$1.33	<$0.01	0.2%	--	--	--
Discretionary choices - other	$314.39	$2.44	24.9%	--	--	--	$296.70	$2.21	23.9%	--	--	--	$167.87	$0.36	20.7%	--	--	--
Alcoholic drinks	$114.81	$0.63	9.1%	--	--	--	$115.18	$0.66	9.3%	--	--	--	$85.72	$1.54	10.6%	--	--	--
Take-away foods	$217.35	$1.64	17.2%	--	--	--	$265.96	$1.79	21.4%	--	--	--	$184.22	$2.13	22.7%	--	--	--
Sugar sweetened soft drinks	$40.29	$0.46	3.2%	--	--	--	$54.73	$0.63	4.4%	--	--	--	$16.41	<$0.01	2.0%	--	--	--
**Total cost**	**$1,263.31**	**$8.65**	**100%**	**$1,078.42**	**$14.07**	**100%**	**$1,241.20**	**$7.89**	**100%**	**$1,078.42**	**$14.07**	**100%**	**$809.93**	**$2.66**	**100%**	**$743.17**	**$2.43**	**100%**
Healthy foods and drinks	$576.47	$6.17	45.6%	$1,078.42	$14.07	100%	$508.62	$5.12	41.0%	$1,078.42	$14.07	100%	$355.72	$0.87	43.9%	$743.17	$2.43	100%
Discretionary foods and drinks	$686.83	$3.40	54.4%	--	--	--	$732.58	$3.54	59.0%	--	--	--	$454.21	$2.49	56.1%	--	--	--

**Affordability of diets**												
**Income amount (A$)**	**Broader population household**				**Welfare dependent household**							
	**Habitual Diet – branded products**		**Recommended Diet – branded products**		**Habitual Diet – branded products**		**Recommended Diet – branded products**		**Habitual Diet – cheapest products**		**Recommended Diet – cheapest products**	
	**Affordability**	**Std error**	**Affordability**	**Std error**	**Affordability**	**Std error**	**Affordability**	**Std error**	**Affordability**	**Std error**	**Affordability**	**Std error**
September 2023 Welfare only household income $3,400 per fortnight	37.16%	0.25%	31.72%	0.41%	36.51%	0.23%	31.72%	0.41%	23.82%	0.08%	21.86%	0.07%

Table S15: Diet cost and affordability by food group and food group components of habitual and recommended diets, for a two adult, two children household (Household B) in Greater Brisbane, September 2023

**Mean food group and food group component costs per fortnight for Greater Brisbane**																		
**Food group and food group component**	**Broader population household**						**Welfare dependent household**											
	**Habitual Diet – branded products**			**Recommended Diet – branded products**			**Habitual Diet – branded products**			**Recommended Diet – branded products**			**Habitual Diet – cheapest products**			**Recommended Diet – cheapest products**		
	**Cost (A$)**	**Std Error (A$)**	**Proportion of total diet cost (%)**	**Cost (A$)**	**Std Error (A$)**	**Proportion of total diet cost (%)**	**Cost (A$)**	**Std Error (A$)**	**Proportion of total diet cost (%)**	**Cost (A$)**	**Std Error (A$)**	**Proportion of total diet cost (%)**	**Cost (A$)**	**Std Error (A$)**	**Proportion of total diet cost (%)**	**Cost (A$)**	**Std Error (A$)**	**Proportion of total diet cost (%)**
Water	$21.88	$0.54	2.4%	$21.88	$0.54	2.8%	$14.40	$0.35	1.6%	$21.88	$0.54	2.8%	$4.99	<$0.01	0.8%	$7.58	<$0.01	1.4%
Fruit	$69.47	$1.31	7.5%	$98.94	$4.03	12.9%	$58.09	$1.01	6.3%	$98.94	$4.03	12.9%	$44.58	$0.26	7.5%	$94.98	$1.30	18.0%
Veg. & Legumes	$53.20	$0.64	5.7%	$131.50	$1.85	17.1%	$48.96	$0.52	5.4%	$131.50	$1.85	17.1%	$39.80	$0.20	6.7%	$100.57	$0.51	19.1%
Grains & Cereals	$56.31	$0.83	6.1%	$136.46	$2.22	17.8%	$47.96	$0.74	5.2%	$136.46	$2.22	17.8%	$24.59	$0.16	4.1%	$57.07	$0.17	10.8%
Meats, nuts, seeds, egg & alternatives	$118.21	$1.38	12.7%	$224.15	$2.36	29.2%	$104.75	$1.20	11.4%	$224.15	$2.36	29.2%	$83.56	$0.40	14.0%	$176.37	$0.76	33.4%
Milk, yoghurt, cheese & alternatives	$66.90	$0.58	7.2%	$142.18	$1.31	18.5%	$67.05	$0.57	7.3%	$142.18	$1.31	18.5%	$44.64	$0.25	7.5%	$84.63	$0.60	16.0%
Unsaturated oils & spreads	$1.98	$0.03	0.2%	$12.63	$0.17	1.6%	$2.49	$0.04	0.3%	$12.63	$0.17	1.6%	$1.15	$0.03	0.2%	$6.52	$0.05	1.24
Artificially sweetened soft drink	$7.11	$0.08	0.8%	--	--	--	$4.18	$0.05	0.5%	--	--	--	$1.26	<$0.01	0.2%	--	--	--
Discretionary choices - other	$221.61	$1.87	23.8%	--	--	--	$209.79	$1.69	22.9%	--	--	--	$117.67	$0.24	19.7%	--	--	--
Alcoholic drinks	$93.92	$0.49	10.1%	--	--	--	$92.90	$0.54	10.2%	--	--	--	$70.95	$1.46	11.9%	--	--	--
Take-away foods	$183.07	$1.36	19.7%	--	--	--	$215.89	$1.43	23.6%	--	--	--	$150.59	$1.70	25.2%	--	--	--
Sugar sweetened soft drinks	$35.80	$0.41	3.9%	--	--	--	$48.55	$0.56	5.3%	--	--	--	$14.55	<$0.01	2.4%	--	--	--
**Total cost**	**$929.46**	**$6.22**	**100%**	**$767.76**	**$10.00**	**100%**	**$915.01**	**$5.65**	**100%**	**$767.76**	**$10.00**	**100%**	**$598.32**	**$2.21**	**100%**	**$527.72**	**$1.70**	**100%**
Healthy foods and drinks	$395.06	$4.27	42.5%	$767.76	$10.00	100%	$347.88	$3.47	38.0%	$767.76	$10.00	100%	$244.56	$0.61	40.9%	$527.72	$1.70	100%
Discretionary foods and drinks	$534.41	$2.70	57.5%	--	--	--	$567.14	$2.79	62.0%	--	--	--	$353.76	$2.12	59.1%	--	--	--

**Affordability of diets**												
**Income amount (A$)**	**Broader population household**				**Welfare dependent household**							
	**Habitual Diet – branded products**		**Recommended Diet – branded products**		**Habitual Diet – branded products**		**Recommended Diet – branded products**		**Habitual Diet – cheapest products**		**Recommended Diet – cheapest products**	
	**Affordability**	**Std error**	**Affordability**	**Std error**	**Affordability**	**Std error**	**Affordability**	**Std error**	**Affordability**	**Std error**	**Affordability**	**Std error**
September 2023 Welfare only household income $2,218 per fortnight	41.90%	0.28%	34.61%	0.45%	41.25%	0.25%	34.61%	0.45%	26.97%	0.10%	23.79%	0.08%

Table S16: Diet cost and affordability by food group and food group components of habitual and recommended diets, for an adult female, two children household (Household C) in Greater Brisbane, September 2023

**Mean food group and food group component costs per fortnight for Greater Brisbane**																		
**Food group and food group component**	**Broader population household**						**Welfare dependent household**											
	**Habitual Diet – branded products**			**Recommended Diet – branded products**			**Habitual Diet – branded products**			**Recommended Diet – branded products**			**Habitual Diet – cheapest products**			**Recommended Diet – cheapest products**		
	**Cost (A$)**	**Std Error (A$)**	**Proportion of total diet cost (%)**	**Cost (A$)**	**Std Error (A$)**	**Proportion of total diet cost (%)**	**Cost (A$)**	**Std Error (A$)**	**Proportion of total diet cost (%)**	**Cost (A$)**	**Std Error (A$)**	**Proportion of total diet cost (%)**	**Cost (A$)**	**Std Error (A$)**	**Proportion of total diet cost (%)**	**Cost (A$)**	**Std Error (A$)**	**Proportion of total diet cost (%)**
Water	$13.53	$0.33	2.2%	$13.53	$0.33	2.4%	$9.39	$0.23	1.5%	$13.53	$0.33	2.4%	$3.25	<$0.01	0.8%	$4.69	<$0.01	1.2%
Fruit	$52.27	$1.01	8.5%	$73.57	$2.99	13.1%	$43.12	$0.69	6.8%	$73.57	$2.99	13.1%	$32.62	$0.17	8.2%	$70.62	$0.96	18.2%
Veg. & Legumes	$33.36	$0.41	5.4%	$94.38	$1.38	16.8%	$29.99	$0.31	4.8%	$94.38	$1.38	16.8%	$25.05	$0.12	6.3%	$73.31	$0.38	18.9%
Grains & Cereals	$40.16	$0.61	6.5%	$101.43	$1.66	18.0%	$35.68	$0.54	5.7%	$101.43	$1.66	18.0%	$18.93	$0.10	4.7%	$42.25	$0.13	10.9%
Meats, nuts, seeds, egg & alternatives	$73.64	$0.91	11.9%	$164.68	$1.75	29.2%	$65.92	$0.78	10.4%	$164.68	$1.75	29.2%	$51.67	$0.25	12.9%	$129.15	$0.57	33.3%
Milk, yoghurt, cheese & alternatives	$47.55	$0.40	7.7%	$108.64	$1.00	19.3%	$48.13	$0.40	7.6%	$108.64	$1.00	19.3%	$31.73	$0.18	7.9%	$64.62	$0.46	16.6%
Unsaturated oils & spreads	$1.44	$0.02	0.2%	$6.94	$0.09	1.2%	$1.76	$0.03	0.3%	$6.94	$0.09	1.2%	$0.80	$0.02	0.2%	$3.58	$0.03	0.9%
Artificially sweetened soft drink	$4.22	$0.05	0.7%	--	--	--	$1.67	$0.02	0.3%	--	--	--	$0.50	<$0.01	0.1%	--	--	--
Discretionary choices - other	$165.09	$1.40	26.8%	--	--	--	$155.53	$1.26	24.6%	--	--	--	$84.32	$0.16	21.1%	--	--	--
Alcoholic drinks	$27.83	$0.19	4.5%	--	--	--	$38.17	$0.42	6.0%	--	--	--	$29.47	$0.19	7.4%	--	--	--
Take-away foods	$131.91	$0.97	21.4%	--	--	--	$161.52	$1.04	25.6%	--	--	--	$109.21	$1.27	27.3%	--	--	--
Sugar sweetened soft drinks	$25.67	$0.30	4.2%	--	--	--	$40.53	$0.47	6.4%	--	--	--	$12.15	<$0.01	3.0%	--	--	--
**Total cost**	**$616.67**	**$4.37**	**100%**	**$563.17**	**$7.35**	**100%**	**$631.41**	**$4.02**	**100%**	**$563.17**	**$7.35**	**100%**	**$399.71**	**$1.26**	**100%**	**$388.23**	**$1.26**	**100%**
Healthy foods and drinks	$266.16	$2.97	43.2%	$563.17	$7.35	100%	$235.66	$2.32	37.3%	$563.17	$7.35	100%	$164.56	$0.37	41.2%	$388.23	$1.26	100%
Discretionary foods and drinks	$350.50	$1.93	56.8%	--	--	--	$395.75	$2.12	62.7%	--	--	--	$235.15	$1.33	58.8%	--	--	--

**Affordability of diets**												
**Income amount (A$)**	**Broader population household**				**Welfare dependent household**							
	**Habitual Diet – branded products**		**Recommended Diet – branded products**		**Habitual Diet – branded products**		**Recommended Diet – branded products**		**Habitual Diet – cheapest products**		**Recommended Diet – cheapest products**	
	**Affordability**	**Std error**	**Affordability**	**Std error**	**Affordability**	**Std error**	**Affordability**	**Std error**	**Affordability**	**Std error**	**Affordability**	**Std error**
September 2023 Welfare only household income $1,901 per fortnight	32.44%	0.23%	29.62%	0.39%	33.21%	0.21%	29.62%	0.39%	21.03%	0.07%	20.42%	0.07%

Table S17: Diet cost and affordability by food group and food group components of habitual and recommended diets, for an older retired couple household (Household D) in Greater Brisbane, September 2023

**Mean food group and food group component costs per fortnight for Greater Brisbane**																		
**Food group and food group component**	**Broader population household**						**Welfare dependent household**											
	**Habitual Diet – branded products**			**Recommended Diet – branded products**			**Habitual Diet – branded products**			**Recommended Diet – branded products**			**Habitual Diet – cheapest products**			**Recommended Diet – cheapest products**		
	**Cost (A$)**	**Std Error (A$)**	**Proportion of total diet cost (%)**	**Cost (A$)**	**Std Error (A$)**	**Proportion of total diet cost (%)**	**Cost (A$)**	**Std Error (A$)**	**Proportion of total diet cost (%)**	**Cost (A$)**	**Std Error (A$)**	**Proportion of total diet cost (%)**	**Cost (A$)**	**Std Error (A$)**	**Proportion of total diet cost (%)**	**Cost (A$)**	**Std Error (A$)**	**Proportion of total diet cost (%)**
Water	$3.81	$0.09	1.0%	$3.81	$0.09	1.0%	$1.95	$0.05	0.5%	$3.81	$0.09	1.0%	$0.67	<$0.01	0.3%	$1.32	<$0.01	0.5%
Fruit	$41.19	$0.66	10.5%	$50.74	$2.07	13.8%	$38.15	$0.56	10.1%	$50.74	$2.07	13.8%	$28.41	$0.17	11.3%	$48.71	$0.67	19.0%
Veg. & Legumes	$32.62	$0.39	8.3%	$61.92	$0.90	16.9%	$31.52	$0.39	8.4%	$61.92	$0.90	16.9%	$24.81	$0.16	9.8%	$47.79	$0.25	18.7%
Grains & Cereals	$29.17	$0.50	7.5%	$43.88	$0.70	12.0%	$31.24	$0.58	8.3%	$43.88	$0.70	12.0%	$13.48	$0.07	5.3%	$19.39	$0.05	7.6%
Meats, nuts, seeds, egg & alternatives	$66.54	$0.77	17.0%	$97.91	$1.04	26.7%	$60.21	$0.68	16.0%	$97.91	$1.04	26.7%	$46.69	$0.21	18.5%	$74.48	$0.32	29.1%
Milk, yoghurt, cheese & alternatives	$27.22	$0.23	7.0%	$102.89	$0.95	28.0%	$25.66	$0.21	6.8%	$102.89	$0.95	28.0%	$16.64	$0.08	6.6%	$61.35	$0.43	24.0%
Unsaturated oils & spreads	$1.55	$0.03	0.4%	$5.72	$0.08	1.6%	$1.55	$0.03	0.4%	$5.72	$0.08	1.6%	$0.70	$0.02	0.3%	$2.96	$0.02	1.2%
Artificially sweetened soft drink	$1.55	$0.02	0.4%	--	--	--	$1.14	$0.01	0.3%	--	--	--	$0.34	<$0.01	0.1%	--	--	--
Discretionary choices - other	$86.66	$0.54	22.2%	--	--	--	$87.25	$0.55	23.2%	--	--	--	$54.66	$0.19	21.6%	--	--	--
Alcoholic drinks	$63.68	$0.40	16.3%	--	--	--	$58.16	$0.32	15.5%	--	--	--	$39.08	$0.61	15.5%	--	--	--
Take-away foods	$32.39	$0.33	8.3%	--	--	--	$34.76	$0.37	9.2%	--	--	--	$25.63	$0.39	10.2%	--	--	--
Sugar sweetened soft drinks	$4.58	$0.05	1.2%	--	--	--	$4.47	$0.05	1.2%	--	--	--	$1.34	<$0.01	0.5%	--	--	--
**Total cost**	**$390.96**	**$2.55**	**100%**	**$366.88**	**$4.70**	**100%**	**$376.06**	**$2.50**	**100%**	**$366.88**	**$4.70**	**100%**	**$252.46**	**$0.85**	**100%**	**$255.99**	**$0.84**	**100%**
Healthy foods and drinks	$203.66	$2.06	52.1%	$366.88	$4.70	100%	$191.42	$1.91	50.9%	$366.88	$4.70	100%	$131.74	$0.36	52.2%	$255.99	$0.84	100%
Discretionary foods and drinks	$187.30	$0.68	47.9%	--	--	--	$184.64	$0.74	49.1%	--	--	--	$120.71	$0.68	47.8%	--	--	--

**Affordability of diets**												
**Income amount (A$)**	**Broader population household**				**Welfare dependent household**							
	**Habitual Diet – branded products**		**Recommended Diet – branded products**		**Habitual Diet – branded products**		**Recommended Diet – branded products**		**Habitual Diet – cheapest products**		**Recommended Diet – cheapest products**	
	**Affordability**	**Std error**	**Affordability**	**Std error**	**Affordability**	**Std error**	**Affordability**	**Std error**	**Affordability**	**Std error**	**Affordability**	**Std error**
September 2023 Welfare only household income $1,827 per fortnight	21.39%	0.14%	20.08%	0.26%	20.58%	0.14%	20.08%	0.26%	13.82%	0.05%	14.01%	0.05%

Table S18: Diet cost and affordability by food group and food group components of habitual and recommended diets, for a single adult male household (Household E) in Greater Brisbane, September 2023

**Mean food group and food group component costs per fortnight for Greater Brisbane**																		
**Food group and food group component**	**Broader population household**						**Welfare dependent household**											
	**Habitual Diet – branded products**			**Recommended Diet – branded products**			**Habitual Diet – branded products**			**Recommended Diet – branded products**			**Habitual Diet – cheapest products**			**Recommended Diet – cheapest products**		
	**Cost (A$)**	**Std Error (A$)**	**Proportion of total diet cost (%)**	**Cost (A$)**	**Std Error (A$)**	**Proportion of total diet cost (%)**	**Cost (A$)**	**Std Error (A$)**	**Proportion of total diet cost (%)**	**Cost (A$)**	**Std Error (A$)**	**Proportion of total diet cost (%)**	**Cost (A$)**	**Std Error (A$)**	**Proportion of total diet cost (%)**	**Cost (A$)**	**Std Error (A$)**	**Proportion of total diet cost (%)**
Water	$8.35	$0.21	2.7%	$8.35	$0.21	4.1%	$5.00	$0.12	1.8%	$8.35	$0.21	4.1%	$1.73	<$0.01	0.9%	$2.89	<$0.01	2.1%
Fruit	$17.20	$0.30	5.5%	$25.37	$1.03	12.4%	$14.97	$0.31	5.3%	$25.37	$1.03	12.4%	$11.96	$0.10	6.0%	$24.35	$0.33	17.5%
Veg. & Legumes	$19.84	$0.24	6.3%	$37.12	$0.47	18.1%	$18.97	$0.22	6.7%	$37.12	$0.47	18.1%	$14.75	$0.08	7.4%	$27.26	$0.13	19.5%
Grains & Cereals	$16.15	$0.22	5.2%	$35.04	$0.57	17.1%	$12.28	$0.19	4.3%	$35.04	$0.57	17.1%	$5.65	$0.05	2.8%	$14.82	$0.04	10.6%
Meats, nuts, seeds, egg & alternatives	$44.61	$0.48	14.3%	$59.48	$0.62	29.1%	$38.83	$0.44	13.7%	$59.48	$0.62	29.1%	$31.88	$0.15	16.1%	$47.22	$0.19	33.8%
Milk, yoghurt, cheese & alternatives	$19.36	$0.19	6.2%	$33.54	$0.31	16.4%	$18.92	$0.17	6.7%	$33.54	$0.31	16.4%	$12.91	$0.08	6.5%	$20.01	$0.14	14.3%
Unsaturated oils & spreads	$0.54	$0.01	0.2%	$5.69	$0.08	2.8%	$0.70	$0.01	0.2%	$5.69	$0.08	2.8%	$0.33	$0.01	0.2%	$2.94	$0.02	2.1%
Artificially sweetened soft drink	$2.89	$0.03	0.9%	--	--	--	$2.51	$0.03	0.9%	--	--	--	$0.76	<$0.01	0.4%	--	--	--
Discretionary choices - other	$56.54	$0.48	18.1%	--	--	--	$54.18	$0.44	19.1%	--	--	--	$33.30	$0.11	16.8%	--	--	--
Alcoholic drinks	$66.13	$0.31	21.1%	--	--	--	$54.73	$0.25	19.3%	--	--	--	$41.48	$1.34	20.9%	--	--	--
Take-away foods	$51.16	$0.42	16.3%	--	--	--	$54.36	$0.41	19.2%	--	--	--	$41.40	$0.43	20.8%	--	--	--
Sugar sweetened soft drinks	$10.13	$0.12	3.2%	--	--	--	$8.02	$0.09	2.8%	--	--	--	$2.40	<$0.01	1.2%	--	--	--
**Total cost**	**$312.90**	**$1.90**	**100%**	**$204.59**	**$2.64**	**100%**	**$283.47**	**$1.67**	**100%**	**$204.59**	**$2.64**	**100%**	**$198.57**	**$1.50**	**100%**	**$139.49**	**$0.44**	**100%**
Healthy foods and drinks	$128.94	$1.31	41.2%	$204.59	$2.64	100%	$112.18	$1.16	39.6%	$204.59	$2.64	100%	$79.98	$0.25	40.3%	$139.49	$0.44	100%
Discretionary foods and drinks	$183.96	$0.84	58.8%	--	--	--	$171.29	$0.73	60.4%	--	--	--	$118.59	$1.39	59.7%	--	--	--

**Affordability of diets**												
**Income amount (A$)**	**Broader population household**				**Welfare dependent household**							
	**Habitual Diet – branded products**		**Recommended Diet – branded products**		**Habitual Diet – branded products**		**Recommended Diet – branded products**		**Habitual Diet – cheapest products**		**Recommended Diet – cheapest products**	
	**Affordability**	**Std error**	**Affordability**	**Std error**	**Affordability**	**Std error**	**Affordability**	**Std error**	**Affordability**	**Std error**	**Affordability**	**Std error**
September 2023 Welfare only household income $875 per fortnight	35.74%	0.22%	23.37%	0.30%	32.38%	0.19%	23.37%	0.30%	22.68%	0.17%	15.93%	0.05%

Table S19: Diet cost and affordability by food group and food group components of habitual and recommended diets, for an older single female household (Household F) in Greater Brisbane, September 2023

**Mean food group and food group component costs per fortnight for Greater Brisbane**																		
**Food group and food group component**	**Broader population household**						**Welfare dependent household**											
	**Habitual Diet – branded products**			**Recommended Diet – branded products**			**Habitual Diet – branded products**			**Recommended Diet – branded products**			**Habitual Diet – cheapest products**			**Recommended Diet – cheapest products**		
	**Cost (A$)**	**Std Error (A$)**	**Proportion of total diet cost (%)**	**Cost (A$)**	**Std Error (A$)**	**Proportion of total diet cost (%)**	**Cost (A$)**	**Std Error (A$)**	**Proportion of total diet cost (%)**	**Cost (A$)**	**Std Error (A$)**	**Proportion of total diet cost (%)**	**Cost (A$)**	**Std Error (A$)**	**Proportion of total diet cost (%)**	**Cost (A$)**	**Std Error (A$)**	**Proportion of total diet cost (%)**
Water	$1.79	$0.04	1.0%	$1.79	$0.04	1.0%	$0.66	$0.02	0.4%	$1.79	$0.04	1.0%	$0.23	<$0.01	0.2%	$0.62	<$0.01	0.5%
Fruit	$19.41	$0.31	11.3%	$25.37	$1.03	14.6%	$18.34	$0.28	10.9%	$25.37	$1.03	14.6%	$13.72	$0.09	12.2%	$24.35	$0.33	20.4%
Veg. & Legumes	$15.17	$0.21	8.8%	$30.23	$0.45	17.5%	$14.86	$0.20	8.9%	$30.23	$0.45	17.5%	$11.58	$0.07	10.3%	$23.36	$0.12	19.6%
Grains & Cereals	$12.99	$0.24	7.6%	$17.73	$0.28	10.2%	$13.65	$0.26	8.1%	$17.73	$0.28	10.2%	$5.71	$0.03	5.1%	$7.98	$0.02	6.7%
Meats, nuts, seeds, egg & alternatives	$33.27	$0.39	19.4%	$39.81	$0.44	23.0%	$31.00	$0.35	18.5%	$39.81	$0.44	23.0%	$24.09	$0.12	21.4%	$28.56	$0.13	23.9%
Milk, yoghurt, cheese & alternatives	$13.36	$0.11	7.8%	$55.41	$0.51	32.0%	$12.25	$0.10	7.3%	$55.41	$0.51	32.0%	$7.85	$0.04	7.0%	$33.06	$0.23	27.7%
Unsaturated oils & spreads	$0.68	$0.01	0.4%	$2.86	$0.04	1.7%	$0.69	$0.01	0.4%	$2.86	$0.04	1.7%	$0.31	$0.01	0.3%	$1.48	$0.01	1.2%
Artificially sweetened soft drink	$0.37	<$0.01	0.2%	--	--	--	$0.22	$0.00	0.1%	--	--	--	$0.07	<$0.01	0.1%	--	--	--
Discretionary choices - other	$39.61	$0.25	23.0%	--	--	--	$38.88	$0.25	23.2%	--	--	--	$23.75	$0.08	21.1%	--	--	--
Alcoholic drinks	$20.89	$0.14	12.2%	--	--	--	$22.24	$0.13	13.3%	--	--	--	$14.76	$0.11	13.1%	--	--	--
Take-away foods	$12.49	$0.11	7.3%	--	--	--	$13.32	$0.15	7.9%	--	--	--	$9.85	$0.15	8.8%	--	--	--
Sugar sweetened soft drinks	$1.84	$0.02	1.1%	--	--	--	$1.46	$0.02	0.9%	--	--	--	$0.44	<$0.01	0.4%	--	--	--
**Total cost**	**$171.88**	**$1.22**	**100%**	**$173.20**	**$2.26**	**100%**	**$167.58**	**$1.19**	**100%**	**$173.20**	**$2.26**	**100%**	**$112.34**	**$0.25**	**100%**	**$119.41**	**$0.41**	**100%**
Healthy foods and drinks	$97.05	$1.02	56.5%	$173.20	$2.26	100%	$91.68	$0.94	54.7%	$173.20	$2.26	100%	$63.55	$0.18	56.6%	$119.41	$0.41	100%
Discretionary foods and drinks	$74.83	$0.30	43.5%	--	--	--	$75.90	$0.33	45.3%	--	--	--	$48.80	$0.16	43.4%	--	--	--

**Affordability of diets**												
**Income amount (A$)**	**Broader population household**				**Welfare dependent household**							
	**Habitual Diet – branded products**		**Recommended Diet – branded products**		**Habitual Diet – branded products**		**Recommended Diet – branded products**		**Habitual Diet – cheapest products**		**Recommended Diet – cheapest products**	
	**Affordability**	**Std error**	**Affordability**	**Std error**	**Affordability**	**Std error**	**Affordability**	**Std error**	**Affordability**	**Std error**	**Affordability**	**Std error**
September 2023 Welfare only household income $1,282 per fortnight	13.41%	0.10%	13.52%	0.18%	13.08%	0.09%	13.52%	0.18%	8.77%	0.02%	9.32%	0.03%
